# The correlation between reading and mathematics ability at age twelve has a substantial genetic component

**DOI:** 10.1038/ncomms5204

**Published:** 2014-07-08

**Authors:** Oliver S. P. Davis, Gavin Band, Matti Pirinen, Claire M. A. Haworth, Emma L. Meaburn, Yulia Kovas, Nicole Harlaar, Sophia J. Docherty, Ken B. Hanscombe, Maciej Trzaskowski, Charles J. C. Curtis, Amy Strange, Colin Freeman, Céline Bellenguez, Zhan Su, Richard Pearson, Damjan Vukcevic, Cordelia Langford, Panos Deloukas, Sarah Hunt, Emma Gray, Serge Dronov, Simon C. Potter, Avazeh Tashakkori-Ghanbaria, Sarah Edkins, Suzannah J. Bumpstead, Jenefer M. Blackwell, Elvira Bramon, Matthew A. Brown, Juan P. Casas, Aiden Corvin, Audrey Duncanson, Janusz A. Z. Jankowski, Hugh S. Markus, Christopher G. Mathew, Colin N. A. Palmer, Anna Rautanen, Stephen J. Sawcer, Richard C. Trembath, Ananth C. Viswanathan, Nicholas W. Wood, Ines Barroso, Leena Peltonen, Philip S. Dale, Stephen A. Petrill, Leonard S. Schalkwyk, Ian W. Craig, Cathryn M. Lewis, Thomas S. Price, The Wellcome Trust Case Control Consortium, Peter Donnelly, Robert Plomin, Chris C. A. Spencer

**Affiliations:** 1 Department of Genetics, Evolution and Environment, UCL Genetics Institute, University College London, London WC1E 6BT, UK; 2 King’s College London, Social Genetic and Developmental Psychiatry Centre, Institute of Psychiatry, London SE5 8AF, UK; 3 Wellcome Trust Centre for Human Genetics, University of Oxford, Oxford OX3 7BN, UK; 4 Department of Psychology, University of Warwick, Coventry CV4 7AL, UK; 5 Department of Psychological Sciences, Birkbeck, University of London, London WC1E 7HX, UK; 6 Department of Psychology, Goldsmiths, University of London, London SE14 6NW, UK; 7 Department of Psychology and Neuroscience, University of Colorado Boulder, Boulder, Colorado 80309-0345, USA; 8 Wellcome Trust Sanger Institute, Cambridge CB10 1SA, UK; 9 Telethon Institute for Child Health Research, Centre for Child Health Research, University of Western Australia, Crawley, Western Australia, Australia; 10 Cambridge Institute for Medical Research, University of Cambridge School of Clinical Medicine, Cambridge CB2 0XY, UK; 11 UCL Institute of Cognitive Neuroscience, University College London, London WC1N 3AR, UK; 12 UCL Mental Health Sciences Unit, University College London, London W1W 7EJ, UK; 13 University of Queensland Diamantia Institute, Translational Research Institute, Princess Alexandra Hospital, University of Queensland, Brisbane, Queensland QLD 4102, Australia; 14 Department of Epidemiology and Population Health, London School of Hygiene and Tropical Medicine, London WC1E 7HT, UK; 15 Department of Epidemiology and Public Health, University College London, London WC1E 6BT, UK; 16 Neuropsychiatric Genetics Research Group, Institute of Molecular Medicine, Trinity College Dublin, Dublin 2, Ireland; 17 Molecular and Physiological Sciences, The Wellcome Trust, London NW1 2BE, UK; 18 Centre for Digestive Diseases, Blizard Institute, Queen Mary University of London, London E1 2AT, UK; 19 Wolfson College, Linton Road, Oxford OX2 6UD, UK; 20 Peninsula School of Medicine and Dentistry, Associate Deans Office, John Bull Building, Plymouth PL6 8BU, UK; 21 Clinical Neurosciences, Saint George's University of London, London SW17 0RE, UK; 22 Department of Medical and Molecular Genetics, King’s College London, King’s Health Partners Guy’s Hospital, London SE1 9RT, UK; 23 Biomedical Research Centre, Ninewells Hospital and Medical School, Dundee DD1 9SY, UK; 24 Department of Clinical Neurosciences, University of Cambridge, Addenbrooke’s Hospital, Cambridge CB2 2QQ, UK; 25 NIHR Biomedical Research Centre at Moorfields Eye Hospital NHSFT and UCL Institute of Ophthalmology, London EC1V 2PD, UK; 26 Department of Molecular Neuroscience, Institute of Neurology, University College London, London WC1N 3BG, UK; 27 Department of Speech and Hearing Sciences, University of New Mexico, Albuquerque, New Mexico 87131, USA; 28 Department of Family Science, Institute for Population Research, The Ohio State University, Columbus, Ohio 43210, USA; 29 Institute for Translational Medicine and Therapeutics, University of Pennsylvania School of Medicine, Philadelphia, Pennsylvania 19104-5158, USA; 30 Department of Statistics, University of Oxford, Oxford OX1 3TG, UK; 31 These authors contributed equally to this work; 32 These authors jointly supervised this work

## Abstract

Dissecting how genetic and environmental influences impact on learning is helpful for maximizing numeracy and literacy. Here we show, using twin and genome-wide analysis, that there is a substantial genetic component to children’s ability in reading and mathematics, and estimate that around one half of the observed correlation in these traits is due to shared genetic effects (so-called Generalist Genes). Thus, our results highlight the potential role of the learning environment in contributing to differences in a child’s cognitive abilities at age twelve.

## Abstract (web summary)

Understanding the genetic basis of cognitive traits could aid the development of numeracy and literacy skills in children. Here the authors show that reading and mathematics have a large overlapping genetic component and suggest that a child's learning environment has a key role in creating differences between them.

## Graphical abstract

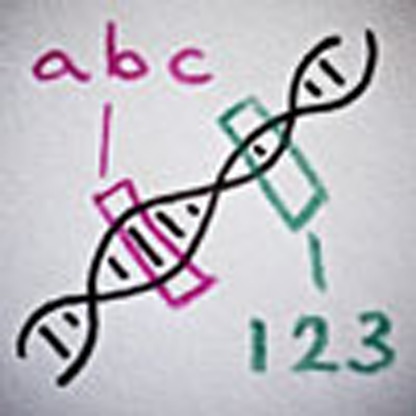

Understanding the aetiology of complex cognitive traits such as reading and mathematics ability is essential for helping children achieve their potential^1^. These traits are highly heritable^2^,^3^ and have been shown to associate with quality of life including wealth and life expectancy^4^,^5^. In spite of their importance and well-established heritability, much remains to be understood about the genetic architecture of cognitive abilities and the genetic component to the correlation between them.

It has been shown^6^,^7^ that population variation in cognitive abilities shares a substantial genetic component with learning difficulties such as dyslexia and dyscalculia (defined here as the low extreme of the distribution^8^). These difficulties affect more than 10% of the population of English-speaking countries^9^, with undiagnosed problems costing economies billions of dollars per year, as well as the less well-documented human cost of missed opportunities. Dyslexia is by far the most frequently diagnosed form of learning difficulty in school-age children^10^, it shows strong stability across childhood and adolescence^10^, and frequently co-occurs with other childhood learning difficulties and psychopathologies^11^,^12^. Although much less is known about dyscalculia, numeracy is as much a requirement as literacy in our increasingly technological world^12^,^13^.

Dyslexia was one of the first traits studied using QTL sib-pair linkage analysis^14^, and although it has been proven to be difficult to identify the genes responsible for these linkages, several candidate genes are under scrutiny^14^,^15^. The first steps towards genome-wide association studies (GWAS) of reading and mathematics ability, using pooled DNA on microarrays, concluded that it is likely that no common genetic variants of large effect influence either trait^16^,^17^. Until recently^1^,^18^,^19^, no common variants associated with the normal range of cognitive traits have been discovered with compelling levels of evidence, although some candidates have been reported.

Here we conduct a GWAS of Reading and Mathematics abilities in a sample of ~3,000 twin pairs. We find no replicable loci with convincing levels of evidence for association, consistent with a substantially polygenic contribution of genetics to these traits. Using bivariate twin- and population-level models, we estimate the heritability and genetic correlation between the two traits. We find a high genetic correlation (around 70%), indicating substantial pleiotropy, and accounting for a large proportion of phenotypic correlation.

## Results

As part of the Wellcome Trust Case-Control Consortium 2 (WTCCC2), in collaboration with the Twins Early Development Study (TEDS), we performed a GWAS using 2,794 unrelated members of monozygotic (MZ) and dizygotic (DZ) twin pairs, measured for their reading and mathematics ability using a combination of web- and phone-based tests at age twelve. The scores were combined across tests and adjusted for age, while gender was used as a covariate in the analyses. Using genotype imputation we performed association analysis for 1,588,650 autosomal markers with reading and mathematics scores separately (see Methods). We followed up the strongest signals of association (*P*_*GWAS*_<5 × 10^−5^; reading, *N*=2,243; mathematics, *N*=2,772) in a further 2,153 individuals, some of whom were co-twins of individuals in the discovery data. One region on chromosome 19 (rs349045) achieved a *P*-value of 9.63 × 10^−9^ (Merlin, *N*=6,061) for reading ability in the joint analysis of discovery and replication data. However, this association failed to replicate using a related phenotype (the Test of Word-Reading Efficiency (TOWRE)—one of four reading tests from the TEDS analysis) in the Avon Longitudinal Study of Parents and Children (ALSPAC, *N*=2,077). The results for the GWAS are shown in Supplementary Figs 1 and 2 and Supplementary Tables 1 and 2. The results from loci previously reported to be associated with reading or mathematics ability or difficulties are reported in Supplementary Table 3.

One explanation for the lack of compelling evidence for association at individual single-nucleotide polymorphisms (SNPs), despite large sample sizes and high heritability estimates, is that the traits studied here are substantially polygenic, with each variant having a small effect. Recent studies have demonstrated that the genetic variants that determine measures of intelligence early and late in life overlap^20^. In our data, standardised reading and mathematics scores show a high correlation, *r*=0.60. This is perhaps unsurprising given that many environmental influences (for example, parenting, schooling and socio-economic factors) will impact on both reading and mathematics ability. Twin studies have also identified a genetic contribution to the correlation^21^. Our data provide the opportunity to clarify the contribution of genetics to the strong correlation in these cognitive abilities using both twin and molecular data in the same sample.

**Figure 1:**
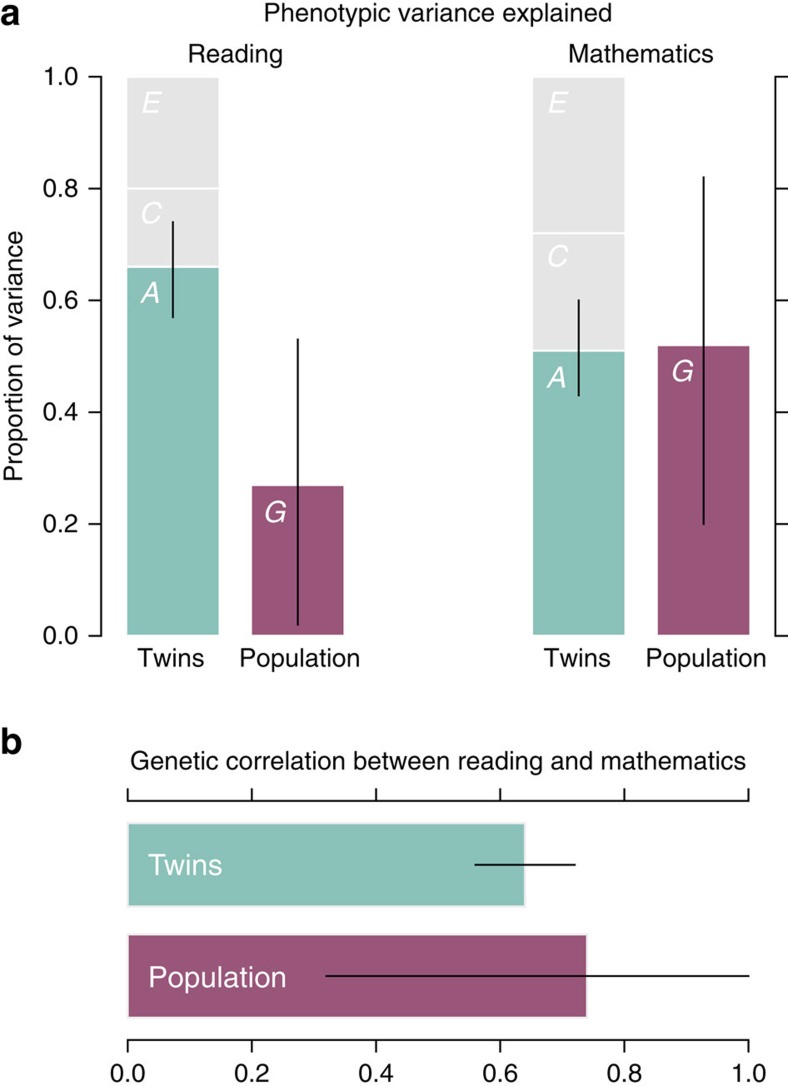
Comparison of twin and population-level analyses. Comparison of estimates of heritability and genetic correlation between reading and mathematics as estimated by twin and population-level models. (**a**) comparison of heritability estimates. *A*, *C* and *E* stand for additive genetic, shared environment and non-shared environment effects, respectively, and *G* denotes the population-level estimate of additive genetic effects. (**b**) comparison of estimates of genetic correlation in reading and mathematics ability from twin (*ρ*_A_) and population-level (*ρ*_G_) models. Bars indicate point estimates (twin model: *N*=2,794 twin pairs; population-level model: *N*=2,221 individuals) with solid black lines indicating 95% confidence intervals.

To investigate the genetic contribution to the correlation, we first fit a bivariate version of the classical twin model using both MZ and DZ twin pairs for whom the reading and mathematics scores were available (Methods and Fig. 1). This method does not use the genotype data, but assumes that genetic relatedness at the variants that affect the traits follows average relatedness of twins (one half for DZ twins and one for MZ twins). The approach estimates the phenotypic covariance explained by additive genetic effects (narrow-sense heritability and correlation), shared environmental influences and non-shared environmental influences across traits. Our analysis estimated the narrow-sense heritability of reading at 0.66 (95% confidence interval (CI): 0.57–0.74), with shared and non-shared environmental contributions of 0.14 (0.06–0.22) and 0.20 (0.18–0.23), respectively. Heritability of mathematics was 0.51 (0.43–0.60), with shared and non-shared environmental estimates of 0.21 (0.14–0.28) and 0.27 (0.25–0.30), respectively. Using the bivariate approach we estimated that the genetic correlation (denoted *ρ*_A_) between reading and mathematics is 0.64 (0.56–0.72), with shared and non-shared environmental correlations of 0.90 (0.67–1.00) and 0.30 (0.24–0.37), respectively.

To exploit the genome-wide data collected as part of this study, we next applied a population-level variance component model to assess polygenic contribution to these traits (see Methods) that bases inference on small differences in allele sharing between individuals who are not closely related^22^. In this model, we estimate the proportion of the phenotypic variance that can be explained by the autosomal SNPs available in the genotyping array data (see Methods) using only the individuals from the GWAS discovery phase (with estimated identity by descent <5%). Using this approach, we estimated the proportion of variance accounted for by the available SNPs as 0.27 for reading (95% CI: 0.02–0.53) and 0.52 (0.20–0.82) for mathematics, with genetic correlation (denoted *ρ*_G_) of 0.74 (0.32–1.00) (Fig. 1 and Supplementary Table 4). By both simulation- and permutation-based approaches we confirmed that the estimated genetic correlation was significantly larger than zero (empirical *P*<0.02, *N*=2,221; see Supplementary Fig. 3).

## Discussion

As discussed elsewhere^23^,^24^, the difference between the twin and population-level models and their underlying assumptions complicates direct comparison of estimated parameters (see Methods). For example, unlike the twin model the population-level approach assumes that all environmental influences are independent among individuals. If there are geographically structured determinates of ability (for example, quality of teaching) that correlate with the genetic differences, then this can inflate population-based estimates of heritability^25^. To address potential confounding by population structure, we fit the model both with and without the leading principal components (PCs) of genetic structure as covariates with similar results (Supplementary Table 4). The population-level approach is also influenced by the coverage of the SNPs used to estimate allele sharing; if a proportion of heritability is due to variants that are not in linkage disequilibrium with typed variants, the population-level model will underestimate heritability. Twin model estimates in principle capture all genetic variation but interpretation of the parameters depends on assumptions regarding the presence of dominance or interaction effects, correlation or interaction between genetics and environment, and putative genetic influences on the shared environmental component. We note that factors affecting additive genetic variance are likely to similarly affect genetic covariance between traits, so that estimates of genetic correlation may be more robust to these effects. The observation that the two different approaches, using different information in the data, estimate a substantial correlation in the genetic component of reading and mathematics ability strongly supports a shared genetic basis.

This observation can be interpreted in at least two ways. First, as a decomposition of the correlation in reading and mathematics ability (see Supplementary Methods), where the twin model estimates that 62% of the observed phenotypic correlation is due to additive genetic factors, and the population-level model estimates that 47% of the observed phenotypic correlation is captured by the available SNPs. Second, by assuming that the genetic variants that affect these traits can be classified into either trait-specific or pleiotropic effects, with similar distribution of effect sizes (see Supplementary Methods), we estimate that at least 10%, and probably around a half, of genetic variants that affect at least one of the traits contribute to both traits. These results suggest substantial pleiotropy, in line with the Generalist Genes Hypothesis^7^.

Our results support previous evidence that common learning abilities and their associated disabilities are unlikely to be affected by common genetic variants of large effect; even with a sample of thousands of individuals and 1,588,650 genetic variants, our most convincing signal of association failed to replicate in an independent sample (although it may still be of interest to future studies). However, we do find suggestive evidence in favour of some previously reported associations (see Supplementary Table 3) for reading ability, most notably rs807701 (*P*_*GWAS*_=0.0084, *N*=2,243) in the *DCDC2* gene, which has been implicated in neuronal development^26^,^27^,^28^. As is the case for other complex traits such as height^29^ larger sample sizes and meta-analyses are needed to pinpoint individual genetic variants^19^. The comparison of our population-level and twin-based variance components analysis, conducted in the same cohort using identical phenotypes, shows that the GWAS data were able to explain a significant proportion of the variance in cognitive abilities (Fig. 1). This is particularly true for mathematics, where the population-level model estimate of heritability is very close to the twin model estimate.

Importantly, our analyses show that a substantial proportion of the observed correlation in reading and mathematics abilities is due to genetics. If a large proportion of the genetic factors that affect these traits are pleiotropic, then the factors that lead to differences in an individual’s abilities (or disabilities) are relatively more likely to be environmental. Understanding the aetiology of these patterns increases our chances of developing effective learning environments that will help individuals attain the highest level of literacy and numeracy, increasingly important skills in the modern world.

## Methods

### Twins Early Development Study

TEDS recruited over 15,000 families of twins born in England and Wales^30^ in 1994, 1995 and 1996 and the sample remains representative of the UK population^2^ (Supplementary Note 2). Ethical approval for TEDS has been provided by the Institute of Psychiatry ethics committee, reference number 05/Q0706/228. We excluded from the analyses children with severe current medical problems and children who had suffered severe problems at birth or whose mothers had suffered severe problems during pregnancy. We also excluded twins whose zygosity was unknown or uncertain, whose first language was other than English, and included only twins whose parents reported their ethnicity as ‘white’, which is 93% of this UK sample.

At age 12, the TEDS twins participated in web- and telephone-based testing, as described previously^31^. Four measures of reading ability were used: two measures of reading comprehension and a measure of reading fluency presented on the web, and a fourth measure (TOWRE) administered over the telephone. Mathematics ability was assessed using a web-based battery of tests that included questions from three components of mathematics, based on the UK national curriculum. Both the reading and mathematics phenotypes comprised an equally weighted combination of the quantile-normalized scales. For each phenotype, we regressed out the effect of age before further analyses. Further details are provided in Supplementary Note 2.

Phenotypic measurements were available for 2,243 (reading) and 2,772 (maths) of these, with 2,794 samples having at least one measurement and 2,221 samples having both. The sample genotyped on the Immunochip (*N*=2,432) included *N*=2,153 individuals with at least one phenotypic measurement, of which *N*=1,388 were DZ co-twins of individuals in the discovery sample. For analyses, taking into account family structure, we additionally included untyped MZ and DZ co-twins of individuals typed in the discovery or Immunochip phases (*N*=1,737) for a combined sample of *N*=7,323. For twin analyses, to parallel the population-based variance estimates as closely as possible, we used a sub-sample of the full TEDS cohort: the 2,794 informative samples with genome-wide data plus their co-twins, for a sample size of *N*=2,794 twin pairs.

### Avon Longitudinal Study of Parents and Children

ALSPAC recruited more than 14,000 pregnant women in the former Avon area of the UK (around Bristol and Bath), with estimated dates of delivery between April 1991 and December 1992 (ref. 32). Ethical approval for the study was obtained from the ALSPAC Ethics and Law Committee and the Local Research Ethics Committees. The ALSPAC study website contains details of all the data that are available through a fully searchable data dictionary ( http://www.bris.ac.uk/alspac/researchers/data-access/data-dictionary/). The sample used for replication here is a population-representative group of participants who were tested for word-reading efficiency (TOWRE) at the age of 12.5 years. After combining TOWRE scores across two subtests (see Supplementary Note 2) a total of *N*=2,140 samples were available for analysis.

### GWAS genotyping and imputation

Samples were genotyped at the Affymetrix’s service laboratory on the Genome-Wide Human SNP Array 6.0. For all samples passing Affymetrix’s laboratory quality control, raw intensities (from the.CEL files) were renormalized within collections using CelQuantileNorm ( http://sourceforge.net/projects/outmodedbonsai/files/CelQuantileNorm/). These normalized intensities were used to call genotypes with an updated version of the Chiamo software^33^ adapted for Affymetrix 6.0 SNP data.

As is the standard practice for GWAS studies, we excluded sets of individuals whose genome-wide patterns of diversity are outliers compared with the majority of those in the study^34^, and we excluded SNPs for which there is evidence that genotype calls do not provide precise estimates of genotype frequencies. Details of the quality control methods used are published elsewhere^34^,^35^. In total, 465 of 3,665 samples were excluded from the analyses by these criteria (see Supplementary Table 5). Genotypes and phenotypes from the discovery sample will be made available through the European Genome-Phenome Archive ( https://www.ebi.ac.uk/ega/).

To assess relatedness among study individuals, we compared each individual with the 100 individuals they were most closely related to (on the basis of genome-wide levels of allele sharing) and used a hidden Markov model (HMM) to decide, at each position in their genome, whether the two individuals shared 0, 1 or 2 chromosomes identical by descent. We obtained a set of ‘unrelated’ individuals with identity by descent <5% by iteratively removing the member of each pair of putatively related individuals with more missing genotypes. A total of 3,154 individuals were included in subsequent analyses.

In addition to standard SNP filters (Supplementary Table 6), we considered a measure of the statistical information (the IMPUTE info measure) carried by the genotype calls for the underlying allele frequency^36^. SNPs were removed prior to imputation if this information measure was below 0.98 or if the estimated minor allele frequency was below 1%. In total 84,029 (9%) of SNPs were removed by these criteria.

We imputed additional genotypes from a combined reference panel of the 120 CEU trios in HapMap2 and HapMap3 and the common control group of WTCCC2. As an additional quality control step, prior to imputation we re-imputed each typed SNP using IMPUTE version 1 and removed any SNP where the concordance between typed and imputed genotypes was <0.965. We used this high-confidence subset of 736,939 SNPs from the array to impute additional genotypes using IMPUTE2 (refs 36, 37).

IMPUTE2 adopts a two-stage approach using both a haploid reference panel and a diploid reference panel. For the haploid reference panel, we used HapMap2 and HapMap3 SNP data on the 120 unrelated CEU trios; and for the diploid reference, we used a merged set of genotype calls from Affymetrix 6.0 and Illumina 1.2 M genotyping chip typed on 5000 1958 Birth Cohort (58C) and National Blood Service (NBS) individuals forming the common control group of WTCCC2. For association testing we included SNPs with info measure of at least 0.98 (if imputed from HapMap) or 0.9 (if imputed from the WTCCC2 controls) and having an estimated minor allele frequency of at least 1%.

### Immunochip genotyping

Replication samples were typed on the Illumina ‘Immunochip’, a custom chip designed by the Immunochip Consortium and WTCCC2, at the Wellcome Trust Sanger Institute. Bead intensity data were processed and normalized for each sample in BeadStudio. Data for successfully genotyped samples were extracted and genotypes were called using the Illuminus algorithm^38^. Samples and SNPs were subject to similar quality control procedures as described above.

### ALSPAC genotyping

We obtained data for 194 SNPs from the region 48891732-49091732 (NCBI build 36) on chromosome 19 for ALSPAC participants by application to the ALSPAC executive. Details on ALSPAC genotyping and imputation are described elsewhere^39^. Samples were included in the analysis if they had attended the TOWRE test session and completed both parts of the test. *N*=63 individuals who were recorded as having scored zero on either part of the test were removed, leaving a total of *N*=2,077 individuals for analysis.

### Genome-wide association analysis

After quality control, 1,588,650 SNPs were analyzed in the GWAS using SNPTEST ( https://mathgen.stats.ox.ac.uk/genetics_software/snptest/snptest.html), fitting an additive linear model to the data, with sex as a covariate. We used the missing data likelihood score test implemented in SNPTEST to compute *P*-values, and refer to these as *P*_GWAS_ above. SNPs with a *P*-value<5 × 10^−5^ were further analyzed in a sample combining the GWAS and Immunochip participants, along with informative co-twins with phenotype data but no genotypes available (*N*=1,737). To take account of the relatedness in the combined sample, we fitted the association model using Merlin software^40^, again with sex as a covariate. The Merlin software also infers the posterior probabilities of missing genotype information from available pedigree information to increase power. For the region on chromosome 19 we analyzed the ALSPAC data using SNPTEST again with sex as a covariate.

### Variance component analysis

For twin and population-level analyses, we consider a general partitioning of a quantitative phenotype *Y* (either reading or mathematics ability in our study) into five components *Y=A+D+I+C+E*, where *A*, *D* and *I* correspond to additive, dominance and interaction genetic effects over the whole genome, respectively, and *C* and *E* are within-family and individual environmental effects, respectively. We assume that these components are defined to be uncorrelated with each other and thus the phenotypic variance is also partitioned into five components *V*_*Y*_=*V*_*A*_+*V*_*D*_+*V*_*I*_+*V*_*C*_+*V*_*E*_.

### Bivariate twin analysis

We consider the traditional ACE twin model (see Supplementary Methods) assuming that dominance and interaction effects are zero (*D*=*I*=0). To extend the model to bivariate phenotype, we introduce three parameters *ρ*_*A*_, *ρ*_*C*_ and *ρ*_*E*_ to describe the correlation between additive genetic, shared environmental and individual environmental effects, respectively, between reading and mathematics abilities (see Supplementary Methods). We use the model to estimate the variance components *V*_*A*_, *V*_*C*_, *V*_*E*_, and the three correlation parameters. The narrow-sense heritability is then defined as the ratio—*V*_*A*_/*V*_*Y*._

For the twin analyses, standardized residuals correcting for age and sex were used because the age of twins is perfectly correlated across pairs, which means that, unless corrected, variation within each age group at the time of testing would contribute to the correlation between twins and be misrepresented as shared environmental influence. The same applies to the sex of the twins, since MZ twins are always of the same sex. The model was fitted using full information maximum likelihood analysis of raw data in the structural equation modelling R package *OpenMx*^41^, estimating the variance parameters for both phenotypes together with the correlation parameters.

### Bivariate population-level analysis

As opposed to the twin model, the population-level model considers only individuals who are not closely related. The univariate version of this model was recently introduced to study human height^22^,^25^ and subsequently further assessed^24^. The bivariate extension was also recently considered^20^,^42^.

This model decomposes the variance into an additive genetic component (*G*) that is due to the available panel of SNPs, and the residual component which in principle includes *D*, *I*, *C* and *E* as defined previously, together with the part of the additive component A that is not captured by *G*. Thus it can be used for estimating a lower bound for the variance and covariance (or correlation) between the additive genetic components of the two phenotypes (Supplementary Methods). A caveat of this model is that environmental effects that correlate with genetics can act as potential confounders.

As for the twin model, we extend the population-level model to the bivariate case by introducing parameters *ρ*_*G*_ and *ρ*_*ε*_ to describe the correlation between genetic and residual effects between phenotypes (see Supplementary Methods). This model was previously used elsewhere^20^.

For population-level analysis, we included one member of each of those 2,221 twin pairs for which both the reading and the mathematics ability was measured. After quality control 686,458 autosomal SNPs from the Affymetrix array were included in the analysis. We used linear regression to adjust the phenotypes for age, sex and population structure (using 10 PCs) before the variance component analysis. We checked that the programme GCTA^22^ gave very similar results for the variance parameters as our own implementation (see Supplementary Methods).

We implemented a Metropolis-Hastings random walk algorithm to explore the posterior distribution on the parameters (here proportional to the likelihood due to the use of uniform priors, see Supplementary Figs 4 and 5 and Supplementary Methods), and compute credible intervals. Finally, we computed a Bayes factor to quantify the evidence for this model relative to the model where *ρ*_*G*_=0, and used both permutations and simulations to obtain a *P*-value for this model comparison (Supplementary Fig. 5 and Supplementary Methods).

### Interpretation of twin and population-level estimates

Although the twin and population models are similar in spirit, they differ in modelling assumptions and parameter interpretation. Here we list factors that could potentially lead to differences between the model estimates.

Population-level estimates of heritability and genetic correlation take into account only those genetic factors which are tagged by variants present on the genotyping platform. Consequently the population-level model gives a lower-bound estimate of heritability. In particular, we chose not to include genotypes of SNPs on the sex chromosomes in order to help simplify the model and its interpretation. In principle, twin model estimates capture all genetic factors which produce differences between trait values for siblings.

Twin model estimates of narrow-sense heritability may be biased upwards by the presence of dominance or interaction effects^23^. By contrast, because the population-level model employs only distantly related individuals, dominance and interaction effects are expected to be much less important effects.

Twin models take into account only those genetic factors which lead to differences in trait values between siblings: consider an unmeasured environmental variable *S* that depends only on the family (socio-economic status may be one such variable). If *S* is correlated with genetics—for example, through parental genotypes—its effect in the twin model, where *S* is unmodelled, will be to increase the estimated proportion of *C*^43^ and so to act to deflate estimates of genetic contributions. However, since shared environment is not modelled in the population-level model, *S* potentially contributes to the estimated proportion of *G*. In principle, including PCs in the model may help control for this effect. We find little difference in the estimates between the model including and not including PCs (Supplementary Table 4).

More complex effects, including interaction between genetic and environmental influences, could potentially have different effects on the two models. For example, twin model estimates of heritability may be affected by within-family (that is, shared) environmental effects that are more similar between MZ twins than DZ twins^44^,^45^. More generally, environmental effects that correlate or interact with genetics and other factors are not modelled directly^43^,^46^. However, several studies have shown that these assumptions of the twin model are usually reasonable in practice^44^,^46^. Population-level estimates appear to be remarkably robust to deviations from modelling assumptions^24^.

**T**he population-level model assumes a particular dependency between the minor allele frequency at a SNP and the size of the effect of that SNP on the phenotype (see Supplementary Methods). Simulation studies^24^ suggest the model is fairly robust to deviations from this assumption.

## Author contributions

Writing Group: O.S.P.D., G.B., M.P., P.D., R.P. and C.C.A.S. Data analysis: O.S.P.D., G.B., M.P., A.S., T.S.P., C.F., C.B., Z.S., R.P., D.V., P.D., C.C.A.S., K.B.H., M.T., L.S.S. and C.M.L. Sample preparation and genotyping: E.L.M., S.J.D., C.J.C.C., I.W.C., C.L., P.D., S.H., E.G., S.D., S.C.P., A.T.G., S.E. and S.J.B. Phenotype data planning and collection: C.M.A.H., Y.K., N.H., S.A.P. and P.S.D. WTCCC2 Management Committee: P.D., I.B., J.M.B., E.B., M.A.B., J.P.C., A.C., P.D., A.D., J.A.Z.J., H.S.M., C.G.M., C.N.A.P., R.P., A.R., S.J.S., R.C.T., A.C.V. and N.W.W.

## Additional information

**How to cite this article:** Davis O. S. P. *et al.* The correlation between reading and mathematics ability at age twelve has a substantial genetic component *Nat. Commun.* 5:4204 doi: 10.1038/ncomms5204 (2014).

## Supplementary Material

Supplementary Information Supplementary Figures 1-5, Supplementary Tables 1-6, Supplementary Notes 1-2, Supplementary Methods and Supplementary References
