# Supplementary material for: The correlation between reading and mathematics ability at age twelve has a substantial genetic component
Source: Nat Commun. 2014 Jul 8;5:4204. doi: 10.1038/ncomms5204 (PMC4102107; doi:10.1038/ncomms5204)
Supplement: Supplementary Information — Supplementary Figures 1-5, Supplementary Tables 1-6, Supplementary Notes 1-2, Supplementary Methods and Supplementary References [file ncomms5204-s1.pdf]

The correlation between reading and mathematics ability at age twelve has a substantial genetic component

Supplementary Information

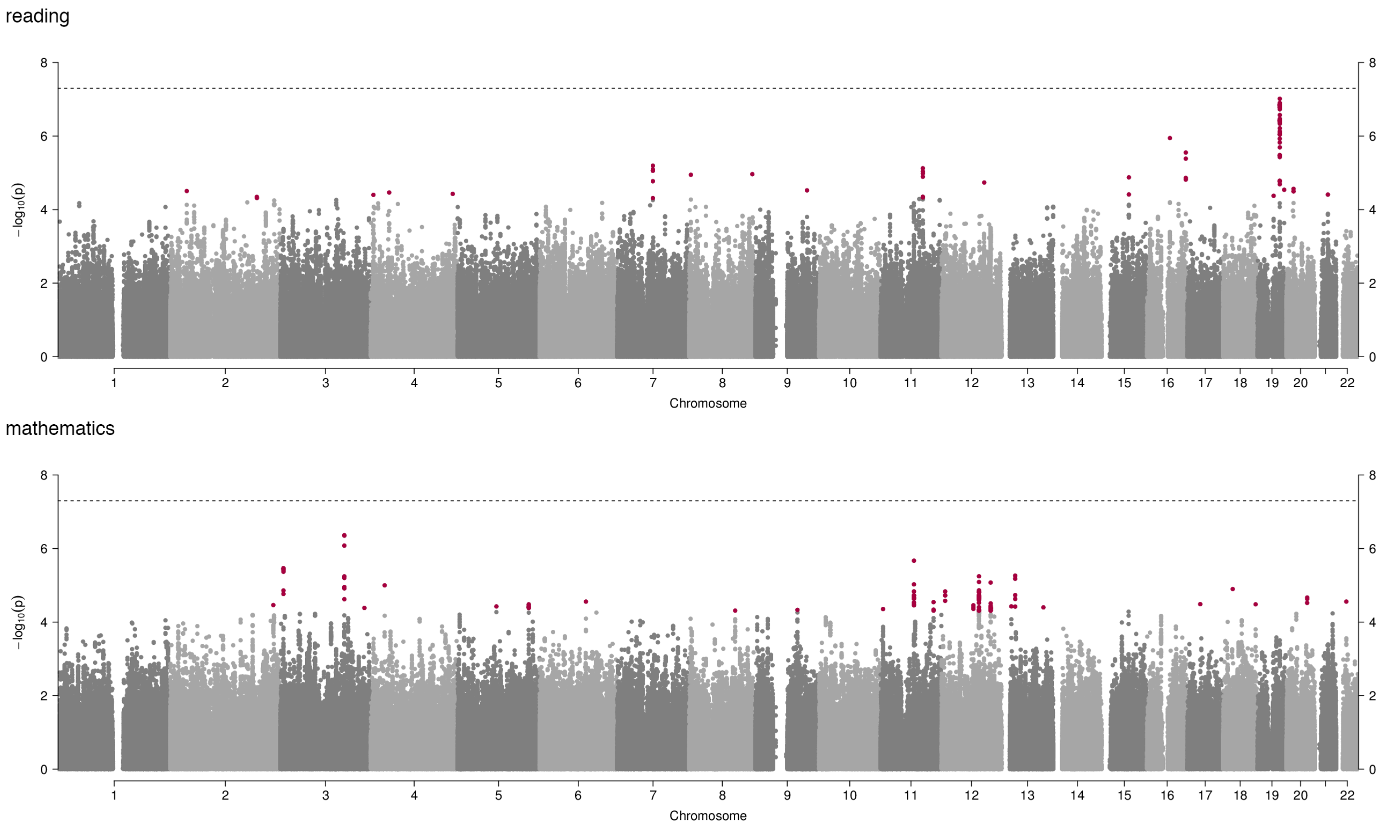

Supplementary Figure 1: Genome-wide Manhattan plots for reading (N=2243) and mathematics (N=2772). Negative $\log_{10}$ of the association P value for each SNP, plotted against genomic position. P-values were computed using the missing data likelihood score test implemented in SNPTEST. Red points represent SNPs with P value less than 5×10^{-5} (i.e. negative $\log_{10}$ P greater than 4.30); these SNPs appear in **Supplementary Table 1** below. The dotted horizontal line represents conventional genome-wide significance of 5×10^{-8} (negative $\log_{10}$ P of 7.30). Clearly visible in the reading plot is the association peak on chromosome 19 that we followed up in our replication sample.

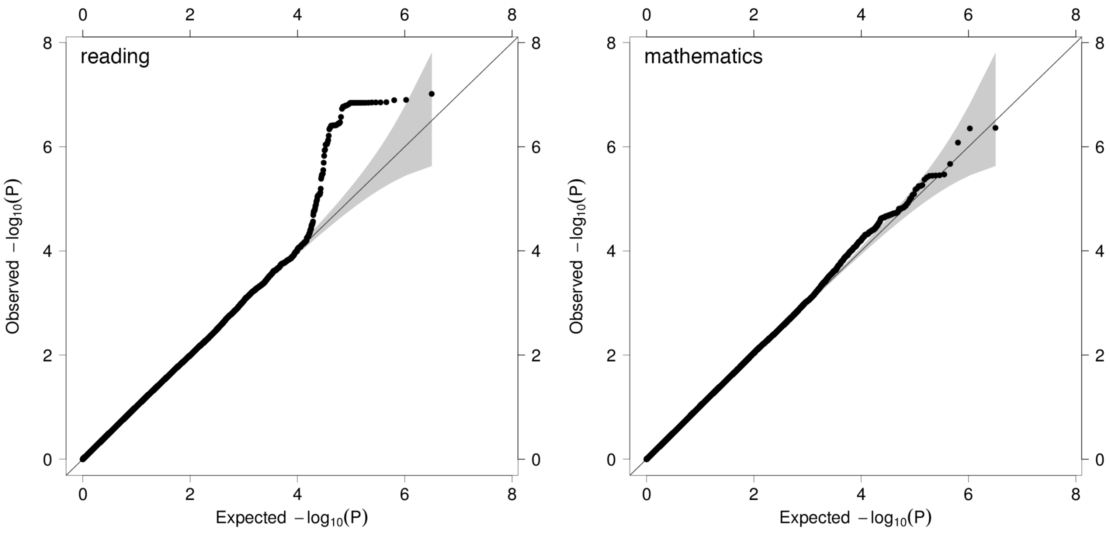

Supplementary Figure 2: Quantile-quantile plots for reading (N=2243) and mathematics (N=2772). Negative $\log_{10}$ of the association P value for each SNP, plotted against the value expected under the null. P-values were computed using the missing data likelihood score test implemented in SNPTEST. The diagonal line is where we would expect the points to fall, on average, if the null hypothesis of no association is true. The gray area is a 95% confidence band on the null. Points falling above the line (and outside the gray band) show a potentially interesting deviation from the null.

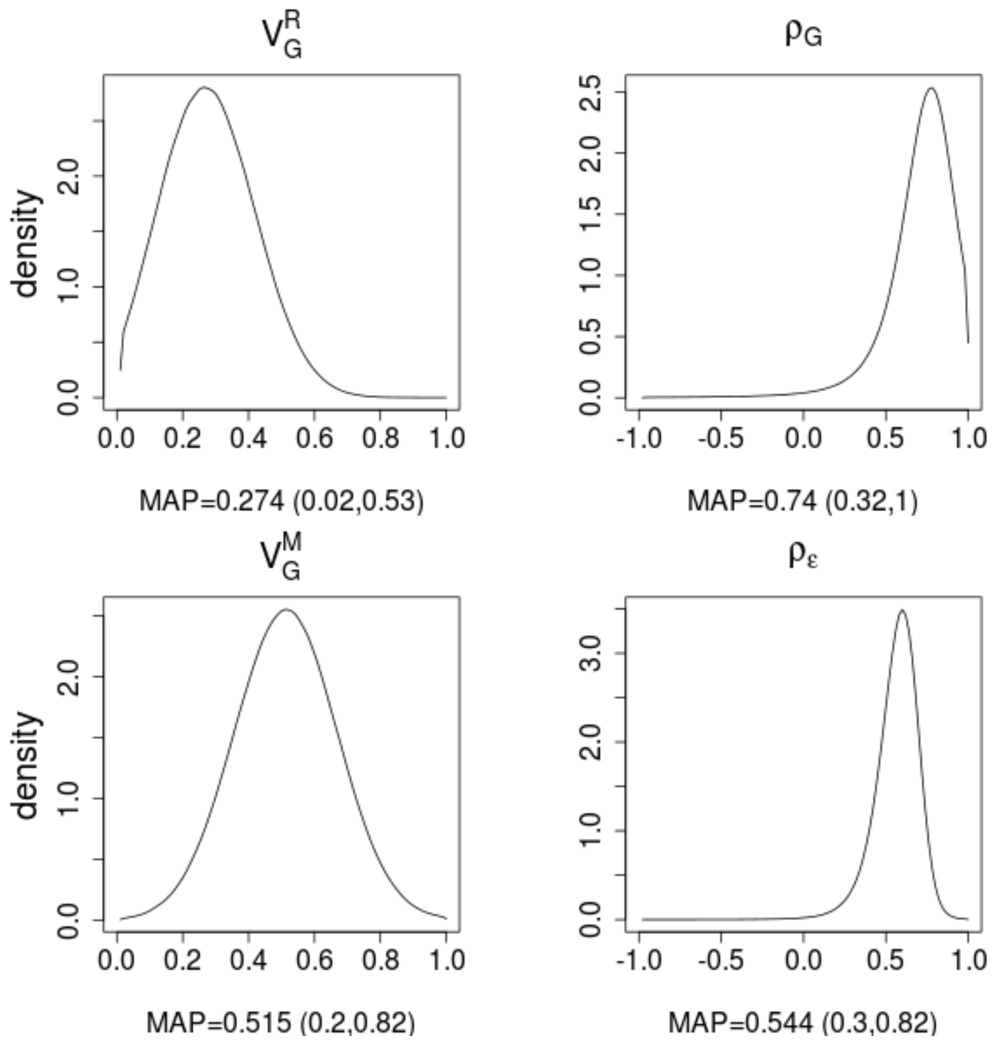

Supplementary Figure 3: One dimensional marginals of the posterior distribution from the population level model. These posterior densities are also proportional to the corresponding likelihood functions (N=2221). Quantities are heritability for reading ($V_G{}^R$) and mathematics ($V_G{}^M$) and correlations of genetic (ρ_G) and environmental components (ρ_ε). Maximum a posteriori point estimates (MAP) as well as 95% credible intervals are below the plots.

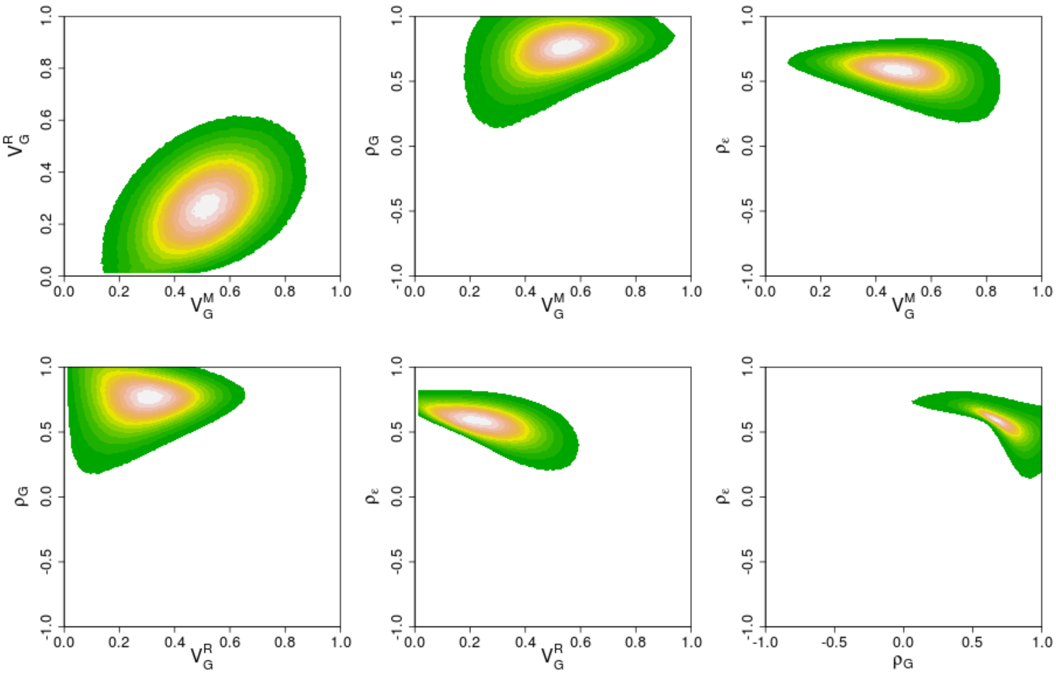

Supplementary Figure 4: Two dimensional marginals of the posterior distribution from the population level model. These posterior densities are also proportional to the corresponding likelihood functions (N=2221). Quantities are heritability for reading ($V_G{}^R$) and mathematics ($V_G{}^M$) and correlations of genetic (ρ_G) and environmental components (ρ_ε). The coloured region contains 95% of the posterior probability with the lightest colour corresponding to the highest value of the density and the yellow stripe showing where the density is halved compared to its maximum value.

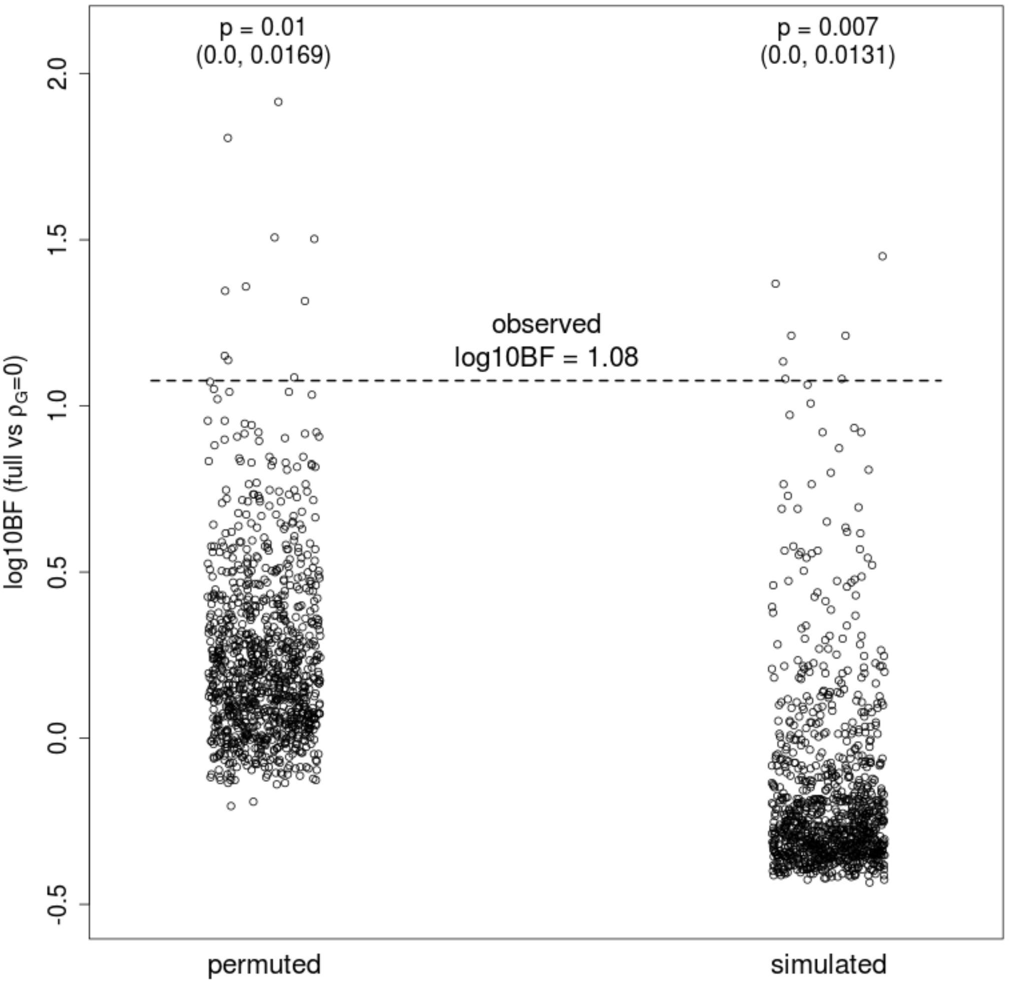

Supplementary Figure 5: Empirical distributions of the log10 Bayes factor. Empirical distributions of the log10 Bayes factor comparing the full model to the model where $\rho_G = 0$, based on 1,000 replications under two data generating processes: permutation and simulation. The observed value (1.08) is shown with a horizontal line. P values and their 95% probability intervals (N=2221) are given at the top of the Figure.

Supplementary Table 1: Strongest associations for reading (P values less than 5×10^{-5})

SNP	Chr[†]	Position[†] (NCBI b36)	Ref allele[†]	RAF	Gene[‡]	Discovery GWAS			GWAS + Immunochip			ALSPAC		
						Beta	SE	P value	Beta	SE	P value	Beta	SE	P value
rs1978723	19	49075640	T	0.50	ZNF404	0.164	0.0307	9.64E-08	-0.106	0.023	3.44E-06	0.0071	0.0353	8.40E-01
rs432454	19	49090671	G	0.49	—	-0.162	0.0306	1.26E-07	0.103	0.023	5.84E-06	-0.0067	0.0352	8.50E-01
rs368089	19	49090924	T	0.50	—	0.162	0.0306	1.28E-07	0.105	0.023	4.16E-06	0.0067	0.0352	8.50E-01
rs8104290	19	49059178	G	0.50	—	-0.161	0.0306	1.39E-07	—	—	—	-0.0057	0.0353	8.72E-01
rs1050054	19	49068521	T	0.50	ZNF404	-0.161	0.0306	1.41E-07	—	—	—	-0.0057	0.0353	8.72E-01
rs4803662	19	49051404	G	0.50	—	-0.161	0.0307	1.41E-07	—	—	—	-0.0057	0.0353	8.72E-01
rs11673020	19	49066471	G	0.50	ZNF404	-0.161	0.0307	1.41E-07	—	—	—	-0.0057	0.0353	8.72E-01
rs397346	19	49090955	G	0.50	—	-0.161	0.0307	1.43E-07	—	—	—	-0.0067	0.0352	8.50E-01
rs372491	19	49093633	G	0.50	—	0.161	0.0307	1.43E-07	—	—	—	0.0093	0.0352	7.91E-01
rs453950	19	49094895	G	0.50	—	0.161	0.0307	1.43E-07	—	—	—	0.0093	0.0352	7.91E-01
rs10406290	19	49070717	T	0.50	ZNF404	-0.161	0.0307	1.43E-07	—	—	—	-0.0057	0.0353	8.72E-01
rs367283	19	49097967	G	0.50	—	-0.161	0.0307	1.43E-07	—	—	—	-0.0093	0.0352	7.91E-01
rs376328	19	49099327	G	0.50	—	-0.161	0.0307	1.43E-07	—	—	—	-0.0093	0.0352	7.91E-01
rs376032	19	49099404	G	0.50	—	-0.161	0.0307	1.43E-07	—	—	—	-0.0093	0.0352	7.91E-01
rs448829	19	49098161	T	0.50	—	0.161	0.0307	1.43E-07	—	—	—	0.0093	0.0352	7.91E-01
rs365556	19	49100039	G	0.50	—	-0.161	0.0307	1.43E-07	—	—	—	-0.0093	0.0352	7.91E-01
rs454813	19	49100813	C	0.50	—	-0.161	0.0307	1.44E-07	—	—	—	-0.0093	0.0352	7.91E-01
rs376457	19	49104597	G	0.50	—	-0.161	0.0307	1.52E-07	—	—	—	-0.0093	0.0352	7.91E-01
rs417400	19	49102686	G	0.50	—	-0.161	0.0306	1.57E-07	—	—	—	-0.0093	0.0352	7.91E-01
rs429027	19	49087329	G	0.50	—	0.160	0.0306	1.62E-07	—	—	—	0.0067	0.0352	8.50E-01
rs430308	19	49092215	G	0.50	—	-0.160	0.0306	1.63E-07	-0.104	0.023	4.99E-06	-0.0067	0.0352	8.50E-01
rs397913	19	49090780	G	0.50	—	-0.160	0.0306	1.70E-07	-0.103	0.023	5.99E-06	-0.0067	0.0352	8.50E-01
rs12977303	19	49069509	G	0.50	ZNF404	0.160	0.0306	1.70E-07	—	—	—	0.0057	0.0353	8.72E-01
rs376069	19	49109135	T	0.50	ZNF45	0.160	0.0307	1.86E-07	—	—	—	0.0093	0.0352	7.91E-01
rs407731	19	49109917	T	0.50	ZNF45	-0.158	0.0307	2.68E-07	0.101	0.023	9.88E-06	-0.0093	0.0352	7.91E-01
rs424729	19	49114642	G	0.50	ZNF45	0.156	0.0307	3.39E-07	—	—	—	0.0174	0.0352	6.20E-01
rs403137	19	49119466	G	0.50	ZNF45	-0.156	0.0307	3.57E-07	—	—	—	-0.0174	0.0352	6.20E-01
rs454559	19	49120960	T	0.50	ZNF45	-0.156	0.0307	3.57E-07	—	—	—	-0.0174	0.0352	6.20E-01
rs388685	19	49110520	G	0.51	ZNF45	0.156	0.0306	3.68E-07	0.099	0.023	1.36E-05	0.0116	0.0353	7.43E-01
rs388706	19	49110533	T	0.50	ZNF45	0.156	0.0306	3.80E-07	—	—	—	0.0116	0.0353	7.43E-01
rs423752	19	49115035	G	0.50	ZNF45	0.155	0.0306	3.87E-07	—	—	—	0.0174	0.0352	6.20E-01
rs378109	19	49113777	T	0.50	ZNF45	0.156	0.0307	3.88E-07	—	—	—	0.0174	0.0352	6.20E-01
rs423320	19	49114822	G	0.50	ZNF45	0.156	0.0307	3.88E-07	—	—	—	0.0174	0.0352	6.20E-01
rs373168	19	49122709	T	0.50	—	-0.156	0.0307	3.88E-07	—	—	—	-0.0174	0.0352	6.20E-01
rs375066	19	49115410	T	0.50	ZNF45	0.156	0.0307	3.90E-07	—	—	—	0.0174	0.0352	6.20E-01
rs379785	19	49119941	T	0.50	ZNF45	0.156	0.0307	3.90E-07	—	—	—	0.0174	0.0352	6.20E-01
rs367741	19	49121020	T	0.50	ZNF45	0.156	0.0307	3.90E-07	—	—	—	0.0174	0.0352	6.20E-01
rs413093	19	49118105	G	0.50	ZNF45	-0.155	0.0306	3.94E-07	—	—	—	-0.0174	0.0352	6.20E-01
rs384329	19	49115821	T	0.50	ZNF45	0.155	0.0306	4.22E-07	0.099	0.023	1.36E-05	0.0174	0.0352	6.20E-01
rs425221	19	49110384	G	0.50	ZNF45	-0.155	0.0306	4.37E-07	0.099	0.023	1.30E-05	-0.0085	0.0352	8.10E-01
rs399098	19	49110664	T	0.49	ZNF45	-0.155	0.0306	4.62E-07	—	—	—	-0.0174	0.0352	6.20E-01
rs417699	19	49109415	G	0.50	ZNF45	-0.153	0.0307	6.13E-07	-0.098	0.023	1.71E-05	-0.0093	0.0352	7.91E-01
rs239938	19	49082145	T	0.47	—	0.154	0.0311	7.40E-07	—	—	—	0.0087	0.0355	8.06E-01
rs171238	19	49071394	C	0.53	ZNF404	-0.153	0.0311	8.12E-07	—	—	—	-0.0077	0.0356	8.28E-01
rs239943	19	49066556	T	0.53	ZNF404	-0.153	0.0311	8.82E-07	—	—	—	-0.0077	0.0356	8.28E-01
rs1073654	19	49103260	T	0.53	—	-0.153	0.0311	9.00E-07	—	—	—	-0.0112	0.0355	7.54E-01
rs1073653	19	49103383	G	0.53	—	-0.153	0.0311	9.00E-07	—	—	—	-0.0112	0.0355	7.54E-01
rs239939	19	49079807	T	0.53	ZNF404	-0.153	0.0311	9.10E-07	—	—	—	-0.0087	0.0355	8.06E-01
rs1125891	16	50401576	T	0.70	—	0.161	0.0331	1.13E-06	—	—	—	—	—	—
rs10426528	19	48991138	T	0.43	LYPD5	0.148	0.0305	1.18E-06	-0.125	0.023	5.69E-08	-0.0390	0.0352	2.68E-01
rs349045	19	48991732	T	0.52	LYPD5	-0.147	0.0305	1.50E-06	-0.132	0.023	9.63E-09	0.0186	0.0352	5.98E-01
rs17656688	19	49116917	T	0.53	ZNF45	-0.148	0.0312	2.02E-06	—	—	—	-0.0202	0.0354	5.69E-01
rs182461	16	85365735	T	0.03	—	-0.399	0.0851	2.80E-06	—	—	—	—	—	—
rs349046	19	48992466	T	0.48	LYPD5	0.143	0.0307	3.28E-06	—	—	—	-0.0186	0.0352	5.98E-01
rs349049	19	48993240	T	0.52	LYPD5	-0.142	0.0306	3.39E-06	—	—	—	0.0186	0.0352	5.98E-01
rs349047	19	48992724	G	0.52	LYPD5	-0.142	0.0306	3.42E-06	—	—	—	0.0186	0.0352	5.98E-01
rs349050	19	48993265	G	0.52	LYPD5	-0.142	0.0307	3.72E-06	—	—	—	0.0186	0.0352	5.98E-01
rs299969	16	85363862	T	0.97	—	0.371	0.0806	4.11E-06	—	—	—	—	—	—
rs1859596	7	78947578	T	0.35	—	-0.143	0.0316	6.39E-06	0.111	0.024	4.36E-06	—	—	—
rs12786407	11	93308703	G	0.76	—	0.156	0.0347	7.52E-06	—	—	—	—	—	—
rs1017040	7	78948774	G	0.65	—	0.141	0.0316	8.18E-06	—	—	—	—	—	—
rs1859598	7	78951930	T	0.65	—	0.141	0.0317	8.25E-06	—	—	—	—	—	—
rs1017039	7	78948728	G	0.65	—	0.141	0.0316	8.29E-06	—	—	—	—	—	—
rs4388396	7	78953651	T	0.35	—	-0.141	0.0316	8.30E-06	—	—	—	—	—	—
rs4727869	7	78929076	T	0.35	—	-0.141	0.0317	8.69E-06	—	—	—	—	—	—
rs2269992	7	78928536	G	0.65	—	0.141	0.0317	8.74E-06	—	—	—	—	—	—
rs4727868	7	78928763	C	0.35	—	-0.140	0.0316	8.75E-06	—	—	—	—	—	—
rs12786631	11	93308818	G	0.76	—	0.154	0.0347	9.19E-06	—	—	—	—	—	—

rs4753523	11	93310749	G	0.24	—	-0.153	0.0347	1.04E-05	—	—	—	—	—	—
rs10505698	8	139368001	G	0.25	FAM135B	0.150	0.0341	1.09E-05	—	—	—	—	—	—
rs2688275	8	3869150	G	0.73	CSMD1	0.146	0.0334	1.13E-05	—	—	—	—	—	—
rs11020610	11	93321480	G	0.76	—	0.151	0.0346	1.27E-05	—	—	—	—	—	—
rs920180	15	60230741	T	0.64	—	-0.140	0.0321	1.33E-05	—	—	—	—	—	—
rs299971	16	85364872	T	0.03	—	-0.375	0.0862	1.38E-05	—	—	—	—	—	—
rs299972	16	85365118	T	0.97	—	0.374	0.0865	1.53E-05	—	—	—	—	—	—
rs11665924	19	48978822	G	0.48	—	0.130	0.0302	1.64E-05	—	—	—	-0.0625	0.0347	7.22E-02
rs1034796	7	78941472	T	0.65	—	0.136	0.0315	1.69E-05	0.106	0.024	9.46E-06	—	—	—
rs3760982	19	48978353	G	0.52	—	-0.130	0.0302	1.69E-05	—	—	—	0.0617	0.0347	7.59E-02
rs3760983	19	48978500	T	0.48	—	0.130	0.0302	1.73E-05	-0.111	0.023	1.12E-06	-0.0613	0.0347	7.76E-02
rs10859872	12	94240125	T	0.78	—	-0.153	0.0357	1.83E-05	—	—	—	—	—	—
rs11666650	19	49100849	T	0.66	—	0.139	0.0326	2.05E-05	—	—	—	-0.0133	0.0375	7.23E-01
rs6135489	20	15662961	T	0.83	MACROD2	-0.163	0.0388	2.72E-05	-0.123	0.029	2.67E-05	—	—	—
rs12983611	19	58815140	G	0.83	—	-0.163	0.0389	2.89E-05	—	—	—	—	—	—
rs4979055	9	113647754	G	0.65	—	-0.130	0.0311	3.00E-05	-0.059	0.023	1.07E-02	—	—	—
rs11124513	2	36472171	G	0.27	CRIM1	0.138	0.0330	3.12E-05	—	—	—	—	—	—
rs2143553	20	15661729	T	0.83	MACROD2	-0.164	0.0393	3.18E-05	—	—	—	—	—	—
rs6043499	20	15664671	T	0.83	MACROD2	-0.163	0.0393	3.19E-05	—	—	—	—	—	—
rs746860	4	40551951	T	0.34	APBB2	0.134	0.0323	3.43E-05	-0.082	0.024	6.75E-04	—	—	—
rs1455659	4	180574108	C	0.40	—	-0.125	0.0304	3.73E-05	0.073	0.023	1.32E-03	—	—	—
rs1569155	15	60247079	G	0.27	—	-0.140	0.0340	3.87E-05	—	—	—	—	—	—
rs2831951	21	28952901	G	0.91	—	0.217	0.0527	3.91E-05	—	—	—	—	—	—
rs4689275	4	5719692	T	0.75	EVC2	-0.141	0.0343	3.98E-05	-0.079	0.026	1.93E-03	—	—	—
rs1433078	19	35532090	T	0.75	—	0.140	0.0343	4.21E-05	0.089	0.026	6.00E-04	—	—	—
rs10501809	11	93305911	G	0.33	—	-0.130	0.0318	4.48E-05	—	—	—	—	—	—
rs10202825	2	190971358	T	0.95	—	0.278	0.0682	4.50E-05	—	—	—	—	—	—
rs1607402	11	93300380	T	0.33	—	-0.130	0.0319	4.67E-05	—	—	—	—	—	—
rs13003362	2	191161263	G	0.93	—	0.249	0.0612	4.84E-05	—	—	—	—	—	—
rs7802883	7	78923199	G	0.65	—	0.129	0.0317	4.86E-05	—	—	—	—	—	—

GWAS N = 2243. GWAS + ImmunoChip N = 6061. ALSPAC N = 2140. RAF: reference allele frequency. [†]Positions and alleles are reported relative to the forward strand of the human reference sequence (NCBI build 36). [‡] SNP annotation by the SCAN database: www.scandb.org P values are two-sided

Supplementary Table 2: Strongest associations for mathematics (P values less than 5×10^{-5})

SNP	Chr†	Position† (NCBI b36)	Ref allele†	RAF	Gene‡	Discovery GWAS			GWAS + Immunochip		
						Beta	SE	P value	Beta	SE	P value
rs982074	3	141102100	T	0.21	—	-0.167	0.0331	4.34E-07	0.104	0.025	2.27E-05
rs13065203	3	141096944	G	0.79	—	0.167	0.0330	4.45E-07	0.104	0.024	2.17E-05
rs12638882	3	141037271	G	0.24	—	-0.157	0.0318	8.32E-07	0.085	0.024	2.96E-04
rs7130795	11	73761867	C	0.45	PGM2L1	0.126	0.0266	2.14E-06	-0.057	0.020	3.78E-03
rs345214	3	6799575	G	0.35	—	0.131	0.0282	3.41E-06	—	—	—
rs345203	3	6794466	G	0.35	—	0.130	0.0281	3.53E-06	—	—	—
rs164028	3	6792722	T	0.64	—	-0.129	0.0278	3.56E-06	-0.078	0.021	1.64E-04
rs164025	3	6800678	G	0.35	—	0.131	0.0283	3.59E-06	—	—	—
rs467166	3	6796681	G	0.65	—	-0.130	0.0280	3.64E-06	-0.079	0.021	1.41E-04
rs463149	3	6796860	G	0.35	—	0.129	0.0280	3.88E-06	-0.078	0.021	1.78E-04
rs466544	3	6796304	G	0.35	—	0.129	0.0281	4.27E-06	-0.078	0.021	1.84E-04
rs11838436	13	30140434	T	0.95	—	0.270	0.0594	5.44E-06	—	—	—
rs1115295	12	82619069	C	0.57	—	0.119	0.0263	5.68E-06	0.073	0.020	2.36E-04
rs1823219	3	141055574	T	0.24	—	-0.147	0.0323	5.71E-06	—	—	—
rs16849528	3	141068129	G	0.77	—	0.145	0.0321	6.30E-06	—	—	—
rs11842552	13	30140235	C	0.95	—	0.264	0.0585	6.61E-06	—	—	—
rs768968	12	82634385	G	0.57	—	0.118	0.0264	8.10E-06	—	—	—
rs3825393	12	108367757	T	0.36	MYO1H	0.123	0.0276	8.40E-06	-0.091	0.021	1.33E-05
rs655719	11	73719889	G	0.69	PGM2L1	-0.126	0.0284	9.43E-06	—	—	—
rs4293757	4	30552307	G	0.24	PCDH7	-0.140	0.0316	1.00E-05	0.065	0.024	6.25E-03
rs4683769	3	141087726	T	0.23	—	-0.141	0.0322	1.11E-05	—	—	—
rs12634902	3	141092976	T	0.77	—	0.141	0.0321	1.21E-05	—	—	—
rs16941631	18	21447583	G	0.96	—	-0.302	0.0691	1.26E-05	-0.167	0.052	1.48E-03
rs4133357	12	82618809	C	0.56	—	0.114	0.0262	1.37E-05	—	—	—
rs345215	3	6799734	T	0.33	—	0.125	0.0286	1.38E-05	—	—	—
rs11057119	12	8121817	G	0.89	NECAP1	-0.185	0.0428	1.47E-05	-0.104	0.032	9.91E-04
rs486758	11	73736580	T	0.69	PGM2L1	-0.123	0.0284	1.47E-05	—	—	—
rs1497343	12	82621269	T	0.44	—	-0.114	0.0263	1.52E-05	—	—	—
rs1497346	12	82621730	T	0.44	—	-0.114	0.0263	1.52E-05	—	—	—
rs11115988	12	82631423	C	0.56	—	0.113	0.0262	1.54E-05	—	—	—
rs8181706	12	82623460	C	0.44	—	-0.113	0.0263	1.55E-05	—	—	—
rs8181725	12	82623471	T	0.56	—	0.113	0.0263	1.55E-05	—	—	—
rs6765699	3	6797675	G	0.33	—	0.123	0.0286	1.72E-05	—	—	—
rs904091	12	82474799	G	0.55	—	0.112	0.0262	1.84E-05	—	—	—
rs9315024	13	30136546	G	0.05	—	-0.260	0.0608	1.85E-05	—	—	—
rs596976	11	73721356	G	0.31	PGM2L1	0.121	0.0283	1.87E-05	—	—	—
rs6488858	12	8120680	T	0.89	NECAP1	-0.185	0.0432	1.88E-05	-0.108	0.032	6.83E-04
rs7132147	12	8121428	T	0.11	NECAP1	0.185	0.0432	1.88E-05	-0.110	0.032	4.97E-04
rs634608	11	73762377	G	0.32	PGM2L1	0.120	0.0281	1.90E-05	—	—	—
rs11236087	11	73771786	T	0.32	PGM2L1	0.120	0.0280	1.90E-05	—	—	—
rs12231426	12	82623969	G	0.44	—	-0.113	0.0263	1.92E-05	—	—	—
rs2667477	12	82547519	T	0.45	—	-0.112	0.0262	1.94E-05	—	—	—
rs4882461	12	82566046	T	0.55	—	0.112	0.0262	1.98E-05	—	—	—
rs1497341	12	82531712	G	0.55	—	0.112	0.0262	1.99E-05	—	—	—
rs1563858	12	82656235	T	0.56	—	0.112	0.0263	2.01E-05	—	—	—
rs324782	12	82515610	T	0.55	—	0.112	0.0262	2.01E-05	—	—	—
rs7972155	12	82655565	G	0.56	—	0.112	0.0263	2.04E-05	—	—	—
rs11115984	12	82623776	T	0.56	—	0.112	0.0263	2.07E-05	—	—	—
rs10862732	12	82631379	G	0.44	—	-0.112	0.0263	2.07E-05	—	—	—
rs11116004	12	82654522	T	0.56	—	0.112	0.0263	2.07E-05	—	—	—
rs11115985	12	82623882	C	0.56	—	0.112	0.0263	2.12E-05	—	—	—
rs7106149	11	73758012	G	0.50	PGM2L1	-0.112	0.0264	2.13E-05	—	—	—
rs12227344	12	82624115	G	0.56	—	0.112	0.0263	2.14E-05	—	—	—
rs6094752	20	45689831	T	0.04	NCOA3	-0.283	0.0667	2.15E-05	—	—	—
rs11561341	12	82624852	T	0.56	—	0.112	0.0263	2.16E-05	—	—	—
rs10506894	12	82573774	G	0.45	—	-0.111	0.0262	2.17E-05	—	—	—
rs1063807	11	73723390	G	0.50	PGM2L1	-0.112	0.0264	2.18E-05	—	—	—
rs12228466	12	82572445	G	0.55	—	0.111	0.0262	2.24E-05	—	—	—
rs3193507	11	73722624	T	0.50	PGM2L1	-0.112	0.0264	2.28E-05	—	—	—
rs1511589	12	82585562	G	0.55	—	0.111	0.0262	2.28E-05	—	—	—
rs365870	12	82497868	T	0.45	—	-0.111	0.0262	2.29E-05	—	—	—
rs437375	12	82500144	T	0.45	—	-0.111	0.0262	2.29E-05	—	—	—
rs11832354	12	82630001	T	0.56	—	0.111	0.0263	2.29E-05	—	—	—
rs682068	12	82519152	G	0.45	—	-0.111	0.0262	2.32E-05	—	—	—
rs10793081	11	73770051	T	0.32	PGM2L1	0.118	0.0280	2.34E-05	—	—	—
rs6090704	20	45663611	G	0.95	NCOA3	0.274	0.0648	2.35E-05	—	—	—
rs3885895	13	30136207	G	0.95	—	0.253	0.0599	2.35E-05	—	—	—
rs10935359	3	141093999	G	0.24	—	-0.136	0.0323	2.38E-05	0.085	0.024	3.94E-04

rs10506895	12	82573984	G	0.55	—	0.110	0.0261	2.43E-05	0.073	0.020	2.05E-04
rs1391072	12	82584974	G	0.55	—	0.110	0.0262	2.52E-05	—	—	—
rs11057118	12	8121690	T	0.89	NECAP1	-0.179	0.0426	2.64E-05	-0.106	0.032	7.94E-04
rs5760093	22	22570340	G	0.24	—	0.138	0.0328	2.77E-05	—	—	—
rs9399726	6	102296148	G	0.07	GRIK2	0.227	0.0542	2.78E-05	—	—	—
rs1940037	11	116941305	T	0.27	DSCAML1	-0.125	0.0300	2.87E-05	0.092	0.022	3.00E-05
rs598869	11	73724623	T	0.68	PGM2L1	-0.117	0.0281	2.93E-05	—	—	—
rs324771	12	82522534	T	0.54	—	0.110	0.0263	2.99E-05	—	—	—
rs6090684	20	45536804	G	0.96	—	0.279	0.0669	3.00E-05	—	—	—
rs7954144	12	108500276	G	0.53	MVK	-0.112	0.0268	3.10E-05	-0.079	0.020	8.93E-05
rs11649906	17	28508850	G	0.66	ACCN1	-0.122	0.0293	3.25E-05	—	—	—
rs10898982	11	73770916	G	0.50	PGM2L1	0.109	0.0263	3.26E-05	-0.049	0.020	1.21E-02
rs12953588	18	71943444	T	0.25	—	-0.128	0.0308	3.29E-05	0.084	0.023	2.64E-04
rs9313608	5	156832625	T	0.07	ICHTHYIN	-0.219	0.0527	3.29E-05	0.105	0.039	6.85E-03
rs10498209	2	227245585	T	0.13	—	0.165	0.0399	3.44E-05	-0.095	0.029	1.17E-03
rs10515750	5	156742650	T	0.07	CYFIP2	-0.218	0.0525	3.46E-05	—	—	—
rs7305505	12	70293351	G	0.93	CCDC131	0.222	0.0537	3.49E-05	—	—	—
rs11236088	11	73771991	G	0.68	PGM2L1	-0.116	0.0280	3.51E-05	—	—	—
rs10850380	12	108487202	G	0.53	MMAB	-0.111	0.0268	3.64E-05	—	—	—
rs7298565	12	108421917	G	0.47	UBE3B	0.110	0.0267	3.72E-05	-0.078	0.020	1.07E-04
rs12561035	13	21669301	G	0.75	—	0.127	0.0307	3.74E-05	—	—	—
rs6870122	5	85590381	G	0.97	—	-0.338	0.0820	3.75E-05	-0.205	0.062	9.84E-04
rs11745566	5	156824531	T	0.93	ICHTHYIN	0.218	0.0529	3.76E-05	0.109	0.039	5.52E-03
rs3742303	13	30119106	T	0.04	USPL1	-0.290	0.0703	3.79E-05	—	—	—
rs11749762	5	156834193	T	0.93	ICHTHYIN	0.217	0.0527	3.90E-05	—	—	—
rs7972528	12	82655167	T	0.46	—	-0.108	0.0263	3.93E-05	—	—	—
rs10476050	5	156831452	C	0.93	ICHTHYIN	0.217	0.0529	3.95E-05	—	—	—
rs7967428	12	70375307	G	0.07	TMEM19	-0.225	0.0547	3.96E-05	0.137	0.041	9.41E-04
rs7964021	12	108482028	G	0.47	MMAB	0.110	0.0268	3.96E-05	—	—	—
rs877710	12	108478359	G	0.53	MMAB	-0.110	0.0267	3.97E-05	-0.078	0.020	9.52E-05
rs1932193	13	92363091	C	0.58	—	-0.111	0.0270	3.98E-05	—	—	—
rs888192	12	108479480	G	0.53	MMAB	-0.110	0.0267	4.02E-05	-0.079	0.020	8.87E-05
rs11739062	5	156834279	T	0.07	ICHTHYIN	-0.217	0.0529	4.07E-05	—	—	—
rs7956536	12	108464899	T	0.53	—	-0.109	0.0267	4.10E-05	-0.078	0.020	9.19E-05
rs12493550	3	185235467	G	0.93	HTR3D	-0.224	0.0546	4.12E-05	-0.158	0.041	1.04E-04
rs10476052	5	156833112	C	0.93	ICHTHYIN	0.217	0.0529	4.14E-05	—	—	—
rs9593	12	108479253	T	0.53	MMAB	-0.109	0.0267	4.15E-05	—	—	—
rs11067231	12	108477986	C	0.47	MMAB	0.109	0.0267	4.22E-05	—	—	—
rs7134594	12	108484576	T	0.53	MMAB	-0.110	0.0268	4.27E-05	—	—	—
rs12321904	12	108473611	T	0.53	—	-0.109	0.0267	4.30E-05	—	—	—
rs4766611	12	108472221	G	0.53	—	-0.109	0.0267	4.30E-05	—	—	—
rs1075467	12	108471318	G	0.47	—	0.109	0.0267	4.30E-05	-0.078	0.020	1.01E-04
rs2338104	12	108379551	G	0.53	KCTD10	-0.110	0.0268	4.32E-05	-0.078	0.020	1.02E-04
rs6606732	12	108468944	G	0.47	—	0.109	0.0267	4.33E-05	—	—	—
rs2304269	12	70366539	T	0.93	TMEM19	0.223	0.0547	4.38E-05	—	—	—
rs2241215	12	108409872	G	0.53	UBE3B	-0.109	0.0267	4.42E-05	—	—	—
rs3740996	11	5657857	G	0.87	TRIM5	-0.161	0.0395	4.42E-05	-0.105	0.030	4.03E-04
rs2645975	12	82520258	C	0.46	—	-0.106	0.0261	4.59E-05	0.071	0.020	3.15E-04
rs4936388	11	116945614	T	0.76	DSCAML1	0.127	0.0310	4.60E-05	0.096	0.023	3.05E-05
rs3904534	9	92572115	T	0.23	—	0.130	0.0319	4.66E-05	-0.089	0.024	1.64E-04
rs990646	12	82531365	G	0.54	—	0.106	0.0261	4.69E-05	—	—	—
rs7311187	12	108422290	T	0.53	UBE3B	-0.109	0.0268	4.76E-05	—	—	—
rs2241213	12	108423240	G	0.53	UBE3B	-0.109	0.0268	4.76E-05	—	—	—
rs2241210	12	108434527	G	0.53	UBE3B	-0.109	0.0268	4.76E-05	—	—	—
rs7956788	12	108445345	T	0.47	UBE3B	0.109	0.0268	4.76E-05	—	—	—
rs2241208	12	108447178	G	0.47	UBE3B	0.109	0.0268	4.76E-05	—	—	—
rs2058807	12	108457656	T	0.47	UBE3B	0.109	0.0267	4.77E-05	—	—	—
rs1511582	12	82533914	T	0.54	—	0.106	0.0261	4.78E-05	—	—	—
rs10744826	12	108449895	G	0.53	UBE3B	-0.109	0.0268	4.82E-05	—	—	—
rs1543897	12	108459594	T	0.53	—	-0.109	0.0268	4.82E-05	—	—	—
rs421713	12	82529888	T	0.54	—	0.106	0.0261	4.83E-05	—	—	—
rs2645978	12	82539090	T	0.54	—	0.106	0.0261	4.84E-05	—	—	—
rs2844049	8	101601588	T	0.48	ANKRD46	0.107	0.0264	4.87E-05	-0.061	0.020	1.84E-03
rs2058804	12	108393394	G	0.53	KCTD10	-0.108	0.0267	4.87E-05	—	—	—
rs12789727	11	116940995	C	0.76	DSCAML1	0.127	0.0313	4.88E-05	—	—	—
rs918106	12	108388701	T	0.53	KCTD10	-0.109	0.0267	4.90E-05	—	—	—
rs1397895	12	82544520	T	0.47	—	-0.106	0.0261	4.93E-05	—	—	—

GWAS N = 2772. GWAS + ImmunoChip N = 6061. RAF: reference allele frequency. [†] Positions and alleles are reported relative to the forward strand of the human reference sequence (NCBI build 36). [‡] SNP annotation by the SCAN database: www.scandb.org P values are two-sided

Supplementary Table 3: Discovery sample signal for previously reported associations

Phenotype	SNP	Chromosome[†]	Position[†]	Increaser allele[†]	Gene/Locus[‡]	P Value[*]	Sources
**Reading**	rs1320490	1	225311821	C	CDC42BPA	0.46152	(1)
	rs1000585	2	75676670	G	—	0.86644	(2)
	rs917235	2	75679327	G	—	0.3268	(2)
	rs714939	2	75688615	G	—	0.41911	(2)
	rs1419228	6	24286285	A	DCDC2	0.7303	(3)
	rs807701	6	24381770	A	DCDC2	**0.0083651**	(2,3)
	rs807724	6	24386848	T	DCDC2	0.27601	(2,3)
	rs4504469	6	24696863	C	KIAA0319	0.98796	(3)
	rs761100	6	24740621	C	KIAA0319	0.85446	(2)
	rs9461045	6	24757040	T	TTRAP	0.056067	(2)
	rs2143340	6	24767050	T	TTRAP	0.052167	(2,3)
	rs1842129	6	124879789	C	NKAIN2	**0.0017535**	(1)
	rs1323381	9	110104259	T	—	0.97313	(1)
	rs1160219	11	18701271	C	IGSF22	0.14174	(1)
	rs4754752	11	100730940	T	—	0.60634	(1)
	rs10505938	12	24292224	C	SOX5	0.48834	(1)
	rs10507218	12	106731363	T	—	0.44022	(1)
	rs2192595	14	72404742	G	DPF3	0.90139	(1)
	rs17819126	15	53577202	G	DYX1C1	0.44585	(3)
	rs3743205	15	53577822	C	DYX1C1	0.56785	(3)
	rs12927866	16	80209823	C	CMIP	0.2499	(2)
	rs6564903	16	80211158	C	CMIP	0.85443	(2)
	rs4265801	16	80222553	T	CMIP	0.93167	(2)
	rs16955705	16	80230851	A	CMIP	0.26497	(2)
	rs16973771	16	83018079	T	ATP2C2	0.89641	(2)
	rs2875891	16	83021410	C	ATP2C2	0.70122	(2)
	rs8045507	16	83022078	G	ATP2C2	0.67885	(2)
	rs10485609	20	47099019	G	CSE1L	0.59144	(1)
	rs2409411	21	31842275	C	TIAM1	0.9761	(1)
**Mathematics**	rs12613365	2	191055555	G	FLJ20160	0.46152	(4)
	rs17278234	5	13990476	G	DNAH5	0.59144	(4)
	rs11154532	6	130567068	C	SAMD3	0.48834	(4)
	rs12199332	6	157185419	T	ARID1B	0.14174	(4)
	rs6947045	7	107287183	C	—	0.9761	(4)
	rs2300052	7	107875730	G	NRCAM	0.90139	(4)
	rs11225308	11	101904688	T	MMP7	0.44022	(4)
	rs6588923	11	106125102	T	GUCY1A2	**0.0017535**	(4)
	rs1215603	12	105041007	T	NUAK1	0.60634	(4)
	rs363449	21	29906146	T	GRIK1	0.97313	(4)

N = 2243 for reading, 2772 for mathematics. [†] Positions and alleles are reported relative to the forward strand of the human reference sequence (NCBI build 36). [‡] SNP annotation by the SCAN database: www.scandb.org [*] P values are two-sided because direction of effect is not consistently reported in the original articles. P values <0.05 in bold text

Supplementary Table 4: Parameter estimates and 95% confidence intervals from twin and population-level models. For the twin model, these represent the variance of the additive genetic component of reading (V_A^R) and maths (V_A^M), genetic correlation (ρ_A), shared environmental variance (V_C^R , V_C^M) and correlation (ρ_C), and residual variation (V_E^R, V_E^M)and correlation (ρ_E) between the two traits. Population-level model parameters represent the variance of the additive genetic component due to the available SNPs of reading (V_G^R) and maths (V_G^M), genetic correlation (ρ_G), and residual variation (V_ε^R, V_ε^M)and correlation (ρ_ε) between the two traits.

Model	V_A^R	V_A^M	ρ_A	V_C^R	V_C^M	ρ_C	V_E^R	V_E^M	ρ_E
**Twin model**	0.66 (0.57-0.74)	0.51 (0.43-0.60)	0.64 (0.56-0.72)	0.14 (0.06-0.22)	0.21 (0.14-0.28)	0.90 (0.67-1.00)	0.20 (0.18-0.23)	0.27 (0.25-0.30)	0.30 (0.24-0.37)
	V_G^R	V_G^M	ρ_G				V_ε^R	V_ε^M	ρ_ε
**Population-level model with 10 PCs**	0.27 (0.02-0.53)	0.52 (0.20-0.82)	0.74 (0.32-1.00)				0.73 (0.47-0.98)	0.48 (0.18-0.80)	0.54 (0.30-0.82)
**Population-level model without PCs**	0.31 (0.03-0.58)	0.53 (0.22-0.84)	0.71 (0.32-1.00)				0.69 (0.42-0.97)	0.47 (0.16 - 0.78)	0.55 (0.28 - 0.84)

Supplementary Table 5: Breakdown of number of individuals removed by each step in discovery and Immunochip cohorts. Samples were excluded based on the following criteria. Identity: < 90% concordance with initial sequenom typing; QC: outlying missingness and heterozygosity; intensity: outlying X/Y channel intensity; gender: mismatch between gender modeled from X chromosome intensity and supplied gender; ancestry: outlier on projection of sample onto hapmap PCA; relatedness: > 5% IBD estimated using an HMM approach.

	**Total samples pre/post QC**	**identity**	**QC**	**gender**	**relatedness**	**Ancestry**	**Intensity**
**Discovery**	3665 / 3154	44	377	13	83	59	9
**Immunochip**	2574 / 2432	27	31	-	82	8	-

Supplementary Table 6: Breakdown of number of SNPs excluded prior to imputation. SNPs were excluded based on standard quality control (QC) metrics (proportion of missing calls, minor allele frequency, Hardy-Weinberg *P*-value, and info measure). Further, SNPs were excluded if they were not well imputed by an initial imputation using IMPUTE v1, or if they were duplicate of another SNP on the platform.

	Total autosomal SNPs pre-QC	Excluded based on QC metrics	Excluded using IMPUTE v1	Removed as duplicate	Total included in imputation
**Discovery**	893634	163327	2523	2973	724811

Supplementary Note 1

Membership of Wellcome Trust Case Control Consortium 2

Management Committee

Peter Donnelly (Chair)[1,2], Ines Barroso (Deputy Chair)[3], Jenefer M Blackwell[4, 5], Elvira Bramon[6] , Matthew A Brown[7] , Juan P Casas[8] , Aiden Corvin[9], Panos Deloukas[3], Audrey Duncanson[10], Janusz Jankowski[11], Hugh S Markus[12], Christopher G Mathew[13], Colin NA Palmer[14], Robert Plomin[15], Anna Rautanen[1], Stephen J Sawcer[16], Richard C Trembath[13], Ananth C Viswanathan[17], Nicholas W Wood[18]

Data and Analysis Group

Chris C A Spencer[1], Gavin Band[1], Céline Bellenguez[1], Colin Freeman[1], Garrett Hellenthal[1], Eleni Giannoulatou[1], Matti Pirinen[1], Richard Pearson[1], Amy Strange[1], Zhan Su[1], Damjan Vukcevic[1], Peter Donnelly[1,2]

DNA, Genotyping, Data QC and Informatics Group

Cordelia Langford[3], Sarah E Hunt[3], Sarah Edkins[3], Rhian Gwilliam[3], Hannah Blackburn[3], Suzannah J Bumpstead[3], Serge Dronov[3], Matthew Gillman[3], Emma Gray[3], Naomi Hammond[3], Alagurevathi Jayakumar[3], Owen T McCann[3], Jennifer Liddle[3], Simon C Potter[3], Radhi Ravindrarajah[3], Michelle Ricketts[3], Matthew Waller[3], Paul Weston[3], Sara Widaa[3], Pamela Whittaker[3], Ines Barroso[3], Panos Deloukas[3].

Publications Committee

Christopher G Mathew (Chair)[13], Jenefer M Blackwell[4,5], Matthew A Brown[7], Aiden Corvin[9], Chris C A Spencer[1]

1 Wellcome Trust Centre for Human Genetics, University of Oxford, Roosevelt Drive, Oxford OX3 7BN, UK; 2 Dept Statistics, University of Oxford, Oxford OX1 3TG, UK; 3 Wellcome Trust Sanger Institute, Wellcome Trust Genome Campus, Hinxton, Cambridge CB10 1SA, UK; 4 Telethon Institute for Child Health Research, Centre for Child Health Research, University of Western Australia, 100 Roberts Road, Subiaco, Western Australia 6008; 5 Cambridge Institute for Medical Research, University of Cambridge School of Clinical Medicine, Cambridge CB2 0XY, UK; 6 Department of Psychosis Studies, NIHR Biomedical Research Centre for Mental Health at the Institute of Psychiatry, King's College London and The South London and Maudsley NHS Foundation Trust, Denmark Hill, London SE5 8AF, UK; 7 University of Queensland Diamantina Institute, Brisbane, Queensland, Australia; 8 Dept Epidemiology and Population Health, London School of Hygiene and Tropical Medicine, London WC1E 7HT and Dept Epidemiology and Public Health, University College London WC1E 6BT, UK; 9 Neuropsychiatric Genetics Research Group, Institute of Molecular Medicine, Trinity College Dublin, Dublin 2, Eire; 10 Molecular and Physiological Sciences, The Wellcome Trust, London NW1 2BE; 11 Department of Oncology, Old Road Campus, University of Oxford, Oxford OX3 7DQ, UK , Digestive Diseases Centre, Leicester Royal Infirmary, Leicester LE7 7HH, UK and Centre for Digestive Diseases, Queen Mary University of London, London E1 2AD, UK; 12 Clinical Neurosciences, St George's University of London, London SW17 0RE; 13 King's College London Dept Medical and Molecular Genetics, King's Health Partners, Guy's Hospital, London SE1 9RT, UK; 14 Biomedical Research Centre, Ninewells Hospital and Medical School, Dundee DD1 9SY, UK; 15 King's College London Social, Genetic and Developmental Psychiatry Centre, Institute of Psychiatry, Denmark Hill, London SE5 8AF, UK; 16 University of Cambridge Dept

Supplementary Note 2

Twins Early Development Study (TEDS)

TEDS recruited over 15,000 families of twins born in England and Wales in 1994, 1995 and 1996[5]. Since then, the sample has remained representative of the UK population (ascertained by comparison with census data from the Office of National Statistics[6]). Although twins have the option of participating or not during each phase of data collection, the pairs that do participate remain representative of the larger sample. Informed consent is obtained by post or online consent forms, and a test administrator is then assigned who telephones the family and generally assists and encourages. Ethical approval for TEDS has been provided by the Institute of Psychiatry ethics committee, reference number 05/Q0706/228.

Reading Tests

Reading comprehension. The twins completed an adaptation of the reading comprehension subtest of the Peabody Individual Achievement Test[7], which we will refer to as $PIAT_{rc}$. The $PIAT_{rc}$ assesses literal comprehension of sentences. The sentences were presented individually on the computer screen. Children were required to read each sentence and were then shown four pictures. They had to select the picture that best matched the sentence they had read, using the mouse. All children started with the same items, but an adaptive algorithm modified item order and test discontinuation depending on the performance of the participant. The internet-based adaptation of the $PIAT_{rc}$ contained the same practice items, test items and instructions as the original published test.

As well as the $PIAT_{rc}$, we assessed reading comprehension using the GOAL Formative Assessment in Literacy for Key Stage 3[8]. The GOAL is a test of reading achievement that is linked to the literacy goals for children at Key Stage 3 of the National Curriculum. Questions are grouped into three categories: Assessing Knowledge and Understanding (e.g. identifying information, use of punctuation and syntax), Comprehension (e.g. grasping meaning, predicting consequences), and Evaluation and Analysis (e.g. comparing and discriminating between ideas). Within each category, questions about words, sentences, and short paragraphs are asked. Because we were primarily interested in comprehension skills, we used questions from the two relevant categories, Comprehension, and Evaluation and Analysis, with 20 items from each category. Correct answers were summed to give a total comprehension score.

Reading fluency. Reading fluency was assessed using an adaptation of the Woodcock-Johnson III Reading Fluency Test[9] and the Test of Word Reading Efficiency (TOWRE, Form B)[10]. The Woodcock-Johnson is a measure of reading speed and rate that requires the ability to read and comprehend simple sentences quickly e.g. "A flower grows in the sky? - Yes/No". The online adaptation consists of 98 yes/no statements; children need to indicate yes or no for each statement as quickly as possible. There is a time limit of 3 minutes for this test. Correct answers were summed to give a total fluency score.

The TOWRE, a standardized measure of fluency and accuracy in word reading skills, includes two subtests, each printed on a single sheet: A list of 85 words, called Sight-word Efficiency (SWE), which assesses the ability to read aloud real words; and a list of 54 non-words, called Phonemic Decoding Efficiency (PDE), which assesses the ability to read aloud pronounceable printed nonwords. The child is given 45 seconds to read as many words as possible. Twins were individually assessed by telephone using test stimuli that had been mailed to families in a sealed package with separate instructions that the package should not be opened until the time of testing. The same tester, who was blind to zygosity, assessed both twins in a pair within the same test session.

The TOWRE was administered in the same way to the ALSPAC sample at age 11. This measure was used for replication of the reading associations from the discovery sample.

Mathematics Tests

The items were based on the National Foundation for Educational Research 5-14 Mathematics Series, which is linked closely to curriculum requirements in the UK and the English Numeracy Strategy[11]. The presentation of items was streamed, so that items from different categories were mixed, but the data recording and branching were done within each category. The items were drawn from the following three categories: Understanding Number, Non-Numerical Processes and Computation and Knowledge. Understanding Number requires an understanding of the numerical and algebraic process to be applied when solving problems (e.g., understanding that multiplication and division are inverse operations). For example, “Look at the number 6085. Change the order of the figures around to make the biggest number possible”. Another example is, “Type the missing number in the box: 27 + 27 + 27 + 27 + 27 + 27 = 27 × ___”. Non-Numerical Processes do not rely solely on memory but rather require understanding of non-numerical mathematical processes and concepts, such as rotational or reflective symmetry and other spatial operations. The questions do not have any significant numerical content that pupils need to consider. Three examples follow: “Which is the longest drinking straw? Click on it.” “One of these shapes has corners that are the same. Click on this shape”. “Which card appears the same when turned upside down? Click on it”. Computation and Knowledge assesses the ability to perform straightforward computations using well-rehearsed pencil-and-paper techniques and the ability to recall mathematical facts and terminology. These questions either are mechanistic or rely on memorization of mathematical facts and terminology. The operation is stated or is relatively unambiguous. Three examples follow: “Type in the answer: 76 - 39”. “All 4-sided shapes are called? Click on the answer (Squares, Rectangles, Parallelograms, Kites, Quadrilaterals)”. “Type in the answer: 149 + 785 = ?”. The mathematics battery is described in detail elsewhere[6].

Supplementary Methods

1 Variance component models

We consider a general partitioning of a quantitative phenotype Y (either reading or mathematics ability in our study) into five components $Y = A+D+I+C+E$, where A, D and I correspond to additive, dominance and interaction genetic effects over the whole genome and C and E are shared and individual environmental effects, respectively. We assume that these components are defined to be uncorrelated with each other and thus the phenotypic variance is also partitioned into five components $V_Y = V_A + V_D + V_I + V_C + V_E$ (see e.g. [15] p.85-87, [22]). We apply two models, a twin model and a population level model, to estimate some of these variance components (V_A, V_C and V_E) together with some of their covariances across reading and mathematics ability.

1.1 *Bivariate twin analysis*

We consider the traditional ACE twin model which makes a strong assumption that dominance and interaction effects are zero ($D = I = 0$). Under this model, twins reared together resemble each other only due to the additive effects of shared genetic variants (A) and shared environmental factors (C). In particular, the ACE twin model assumes that the environmental correlations are similar for both MZ and DZ pairs.

To extend the twin model to a bivariate phenotype we introduce three parameters ρ_A, ρ_C and ρ_E to describe the correlation between additive genetic, shared environmental and individual environmental effects between reading and mathematics abilities. More precisely, the bivariate twin model states that the joint distribution of reading (Y^{R}) and mathematics (Y^{M}) is a sum of three components (A, C and E) that follow normal distributions with zero mean and block-wise correlation structures corresponding to families. For the additive genetic component A, the 4×4 block of the covariance matrix corresponding to a family is

$$\text{Var}\begin{bmatrix} A_1^{\mathrm{R}} \\ A_2^{\mathrm{R}} \\ A_1^{\mathrm{M}} \\ A_2^{\mathrm{M}} \end{bmatrix} = \begin{bmatrix} V_A^{\mathrm{R}} & V_A^{\mathrm{R}} & \rho_A S_A & \rho_A S_A \\ V_A^{\mathrm{R}} & V_A^{\mathrm{R}} & \rho_A S_A & \rho_A S_A \\ \rho_A S_A & \rho_A S_A & V_A^{\mathrm{M}} & V_A^{\mathrm{M}} \\ \rho_A S_A & \rho_A S_A & V_A^{\mathrm{M}} & V_A^{\mathrm{M}} \end{bmatrix}, \text{ for MZ twins, and}$$

$$\text{Var}\begin{bmatrix} A_1^{\mathrm{R}} \\ A_2^{\mathrm{R}} \\ A_1^{\mathrm{M}} \\ A_2^{\mathrm{M}} \end{bmatrix} = \begin{bmatrix} V_A^{\mathrm{R}} & \frac{1}{2}V_A^{\mathrm{R}} & \rho_A S_A & \frac{1}{2}\rho_A S_A \\ \frac{1}{2}V_A^{\mathrm{R}} & V_A^{\mathrm{R}} & \frac{1}{2}\rho_A S_A & \rho_A S_A \\ \rho_A S_A & \frac{1}{2}\rho_A S_A & V_A^{\mathrm{M}} & \frac{1}{2}V_A^{\mathrm{M}} \\ \frac{1}{2}\rho_A S_A & \rho_A S_A & \frac{1}{2}V_A^{\mathrm{M}} & V_A^{\mathrm{M}} \end{bmatrix}, \text{ for DZ twins,}$$

where V_A^{R} and V_A^{M} are the additive genetic variance of reading and mathematics, repectively, and $S_A = \sqrt{V_A^{\mathrm{R}} V_A^{\mathrm{M}}}$. The subscripts 1 and 2 index the two individuals of the twin pair.

For the environmental effects, the family-wise covariance matrices are

$$\text{Var}\begin{bmatrix} C_1^{\mathrm{R}} \\ C_2^{\mathrm{R}} \\ C_1^{\mathrm{M}} \\ C_2^{\mathrm{M}} \end{bmatrix} = \begin{bmatrix} V_C^{\mathrm{R}} & V_C^{\mathrm{R}} & \rho_C S_C & \rho_C S_C \\ V_C^{\mathrm{R}} & V_C^{\mathrm{R}} & \rho_C S_C & \rho_C S_C \\ \rho_C S_C & \rho_C S_C & V_C^{\mathrm{M}} & V_C^{\mathrm{M}} \\ \rho_C S_C & \rho_C S_C & V_C^{\mathrm{M}} & V_C^{\mathrm{M}} \end{bmatrix}, \text{ and}$$

$$\text{Var}\begin{bmatrix} E_1^{\mathrm{R}} \\ E_2^{\mathrm{R}} \\ E_1^{\mathrm{M}} \\ E_2^{\mathrm{M}} \end{bmatrix} = \begin{bmatrix} V_E^{\mathrm{R}} & 0 & \rho_E S_E & 0 \\ 0 & V_E^{\mathrm{R}} & 0 & \rho_E S_E \\ \rho_E S_E & 0 & V_E^{\mathrm{M}} & 0 \\ 0 & \rho_E S_E & 0 & V_E^{\mathrm{M}} \end{bmatrix},$$

where V_C^{R} and V_C^{M} are the variance of the shared environmental effects, V_E^{R} and V_E^{M} are the variance of the individual environmental effects (for reading (R) and mathematics (M)) and $S_C = \sqrt{V_C^{\mathrm{R}} V_C^{\mathrm{M}}}$ and $S_E = \sqrt{V_E^{\mathrm{R}} V_E^{\mathrm{M}}}$.

We estimated the variance parameters $(V_A^{\mathrm{R}}, V_A^{\mathrm{M}}, V_C^{\mathrm{R}}, V_C^{\mathrm{M}}, V_E^{\mathrm{R}}, V_E^{\mathrm{M}})$ and the correlations (ρ_A, ρ_C, ρ_E) using maximum likelihood method as implemented in the OpenMx R package (v.1.3.2) [12]. For example, for reading, this allows us to estimate the narrow-sense heritability $V_A^{\mathrm{R}}/V^{\mathrm{R}}$, proportions of shared environmental variance, $V_C^{\mathrm{R}}/V^{\mathrm{R}}$, and individual environmental variance $V_E^{\mathrm{R}}/V^{\mathrm{R}}$, where $V^{\mathrm{R}} = V_A^{\mathrm{R}} + V_C^{\mathrm{R}} + V_E^{\mathrm{R}}$ (and similarly for mathematics).

1.2 Bivariate population level analysis

As opposed to the twin model, the population level model considers unrelated individuals (or more precisely, only very distantly related individuals). For univariate phenotype, this model was introduced by [19] and further explained by [18] and [21]. The bivariate extension was recently considered by [13] and [14].

This model decomposes the variance into an additive genetic component (G) that is due to the available panel of SNPs, and the residual component that includes the previously defined D, I, C and E components together with the part of the additive component A that is not captured by G. Thus the model can be used for estimating a lower bound for the additive genetic variance for both phenotypes ($V_A^{\mathrm{R}}, V_A^{\mathrm{M}}$) and the correlation ($\rho_A$) between the additive genetic components of the two phenotypes.

1.2.1 Univariate linear model

To describe the model for a univariate phenotype, let x_{ij} be the standardised genotype of individual i at SNP j, that is, $x_{ij} = (g_{ij} - 2p_j)/\sqrt{2p_j(1-p_j)}$, where g_{ij} is the minor allele count (0,1 or 2) that i carries at locus j and p_j is the sample average of those allele counts. The linear model assumes that

$$y_i = \mu_i + \sum_j x_{ij}\beta_j + \varepsilon_i, \tag{1}$$

where β_j is the effect of the SNP j, ε_i is the error term for individual i and the sum is over all the SNPs. The term μ_i describes the expected phenotype of individual i after all the non-genetic covariates (such as age, sex and cohort) have been taken into account.

Following [19], we assume that $\beta_j \sim \mathcal{N}(0, V_{\mathrm{g}})$ for each SNP j and $\varepsilon_i \sim \mathcal{N}(0, V_\varepsilon)$ for each individual i. Then the additive genetic variance due to the SNPs,

$$V_G = \mathrm{Var}\left(\sum_j \beta_j^2\right) = LV_{\mathrm{g}},$$

can be estimated by a linear mixed model formulation of the model (1) [19, 21]:

$$\mathbf{Y} = \boldsymbol{\mu} + \mathbf{G} + \boldsymbol{\varepsilon}, \tag{2}$$

where $\boldsymbol{\varepsilon} \sim \mathcal{N}(0, V_\varepsilon \mathbf{I})$ and $\mathbf{G} \sim \mathcal{N}(0, V_G \mathbf{R})$ with the element $\mathbf{R}_{ik}$ of the matrix $\mathbf{R}$ being

$$\mathbf{R}_{ik} = \frac{1}{L}\sum_j x_{ij}x_{kj} = \frac{1}{L}\sum_j \frac{(g_{ij}-2p_j)(g_{kj}-2p_j)}{2p_j(1-p_j)}.$$

Note that since the variance of the effect size distribution, V_{g}, is the same for all standardised SNPs, it follows that the variance of the effect size distribution for an allele at SNP j, $V_{\mathrm{g}}/(2p_j(1-p_j))$, grows as the minor allele frequency p_j decreases. Thus, this models assumes larger per allele effect sizes at rarer SNPs than at common SNPs.

1.2.2 Bivariate linear model

We follow the extension of the linear model to the bivariate case of reading and mathematics phenotypes given by [13]. This corresponds to an instance of the model (2) where

$$\mathbf{Y} = \begin{bmatrix} \mathbf{Y}^{\mathrm{R}} \\ \mathbf{Y}^{\mathrm{M}} \end{bmatrix}, \quad \boldsymbol{\mu} = \begin{bmatrix} \boldsymbol{\mu}^{\mathrm{R}} \\ \boldsymbol{\mu}^{\mathrm{M}} \end{bmatrix}, \quad \mathbf{G} \sim \mathcal{N}(0, \boldsymbol{\Sigma}_G), \text{ and } \boldsymbol{\varepsilon} \sim \mathcal{N}(0, \boldsymbol{\Sigma}_\varepsilon),$$

where

$$\begin{aligned} \boldsymbol{\Sigma}_G &= \left[\begin{array}{c|c} V_G^{\mathrm{R}}\mathbf{R} & \rho_G\sqrt{V_G^{\mathrm{R}}V_G^{\mathrm{M}}}\mathbf{R} \\ \hline \rho_G\sqrt{V_G^{\mathrm{R}}V_G^{\mathrm{M}}}\mathbf{R} & V_G^{\mathrm{M}}\mathbf{R} \end{array}\right] \text{ and} \\ \boldsymbol{\Sigma}_\varepsilon &= \left[\begin{array}{c|c} V_\varepsilon^{\mathrm{R}}\mathbf{I} & \rho_\varepsilon\sqrt{V_\varepsilon^{\mathrm{R}}V_\varepsilon^{\mathrm{M}}}\mathbf{I} \\ \hline \rho_\varepsilon\sqrt{V_\varepsilon^{\mathrm{R}}V_\varepsilon^{\mathrm{M}}}\mathbf{I} & V_\varepsilon^{\mathrm{M}}\mathbf{I} \end{array}\right] \end{aligned}$$

are expressed by $n \times n$ blocks, where n is the number of individuals.

From this model, an estimate of V_G gives a lower bound for the additive genetic variance V_A and thus can be used to estimate a lower bound for the (narrow-sense) heritability. The parameter ρ_G can be interpreted either as the correlation of the additive genetic components (ρ_A) or as the correlation in the genetic effects between the phenotypes $\rho_G = \mathrm{cor}(\beta_j^{\mathrm{R}}, \beta_j^{\mathrm{M}})$. The parameter ρ_ε is the correlation in the error terms between the phenotypes.

1.2.3 Analysis

We analysed this model by using our own implementation as well as by applying publicly available GCTA software v.1.0 [20].

Our implementation. We parameterise the model by six quantities: the phenotypic variances V^{R} and V^{M}, the (lower bounds of narrow-sense) heritabilities V_G^{R} and V_G^{M} and the correlations ρ_G and ρ_ε. (Note that our notation uses genetic variances and heritabilities interchangeably, which is technically correct when the total variance of the phenotype is 1.) We formulate the analysis in the Bayesian framework, and adopt uniform prior distributions for V_G^{R} and V_G^{M} (on the interval $[0, 1]$) and for ρ_G and ρ_ε (on the interval $[-1, 1]$). Since there was very little uncertainty about the total variances V^{R} and V^{M}, we have kept them fixed to their ML-estimates during the analyses reported in this study.

Note that since we are using uniform distributions as priors, our posterior distribution is proportional to the likelihood function, and thus this Bayesian analysis also corresponds to a likelihood analysis.

An implementation of a standard random walk Metropolis-Hastings algorithm (see, e.g., [16]) was possible through a representation of the likelihood function whose computational complexity is linear in the number of individuals.

This is an extension of the likelihood computation described by [17] to the case of bivariate phenotype.

A main motivation for carrying out a sampling based exploration of the posterior instead of a simpler search of the maximum was the interest in the actual shape of the distribution. Thus, we do not need to make normality assumptions when inferring credible intervals for the parameters. The results for the univariate (Supplementary Figure 3) and the bivariate marginal posteriors (Supplementary Figure 4) were computed on the data set described in the main text: 2,221 individuals, 686,458 directly genotyped high quality autosomal SNPs, with phenotypes that were adjusted by regressing out the effects of sex and ten leading principal components of population structure.

As there is large uncertainty about the value of ρ_G (point estimate 0.74, 95% credible interval 0.32 - 1.00), it is of interest to do model comparison between the full model and the null model where $\rho_G = 0$. The Bayes factor (BF) for this model comparison is 12 in favor of the full model. We assessed frequentist properties of this model comparison in two ways. First, we permuted the bivariate phenotypes with respect to the genotype data. This procedure maintains the correlation between the phenotypes, but removes the heritabilities of the phenotypes, and thus also removes any information about the genetic correlation parameter ρ_G. Second, we generated phenotypes for the observed genotype data (summarised by the R matrix) with parameter values $V^{\mathrm{R}} = V^{\mathrm{M}} = 1$, $V_G^{\mathrm{R}} = 0.3$, $V_G^{\mathrm{M}} = 0.5$, $\rho_G = 0$ and $\rho_\varepsilon = 1.0$. The variance parameters were chosen to mimic the observed data and the correlation parameters determined that the correlation between the two phenotypes was 0.60 (as in the observed data), but that none of this correlation was genetic. We then computed the Bayes factors between the full and the null models for 1,000 repetitions of both of these procedures (Supplementary Figure 5). Note that the simulated data provides more information about ρ_G than permuted data, because the former contains a genetic component of the variance whereas the latter lacks it. Therefore the Bayes factor tends to favor the null model more strongly in simulated data than in permuted data, as demonstrated by on average smaller values of BF in simulated data. We conclude that the p-value (probability of getting BF at least as large as the observed one when the null model holds) is less than 0.02 in both cases.

GCTA software. We applied the publicly avilable GCTA software package (v.1.0) [1] [20] to get REML estimates of the variance and correlation parameters. This analysis was done without adjustment for principal components of population structure and used a larger panel of 1.7 million imputed SNPs. The estimated values are 0.27 (se 0.123) for V_G^{R}, 0.48 (se 0.126) for V_G^{M} and 0.71 (se 0.148) for ρ_G. Thus, in practice, the results are the same to our own implementation described above.

Proportion of shared variants. In the main text we state that at least around a half of the genetic basis to reading and mathematics ability is due to shared molecular mechanisms (so-called generalist genes). To explain this, let us

[1] http://www.complextraitgenomics.com/software/gcta/

assume that there are S shared variants that affect both traits and T_{R} and T_{M} variants that affect only reading or mathematics ability, respectively. We further assume that the effect sizes for all these variants follow the normal distribution with zero mean and variance $V_{\mathrm{g}}^{\mathrm{R}}$ (in reading) or $V_{\mathrm{g}}^{\mathrm{M}}$ (in mathematics), and that for each shared variant j, $\mathrm{cor}(\beta_j^{\mathrm{R}}, \beta_j^{\mathrm{M}}) = \rho_S$. The covariance between the genetic components is

$$\mathrm{Cov}(G_i^{\mathrm{R}}, G_i^{\mathrm{M}}) = S\rho_S\sqrt{V_{\mathrm{g}}^{\mathrm{R}}V_{\mathrm{g}}^{\mathrm{M}}}$$

and the genetic variances are $V_G^{\mathrm{R}} = (S + T_{\mathrm{R}})V_{\mathrm{g}}^{\mathrm{R}}$ and $V_G^{\mathrm{M}} = (S + T_{\mathrm{M}})V_{\mathrm{g}}^{\mathrm{M}}$. It follows that the genetic correlation is

$$\rho_G = \frac{S\rho_S}{\sqrt{S+T_{\mathrm{R}}}\sqrt{S+T_{\mathrm{M}}}} = \frac{\rho_S}{\sqrt{1+T_{\mathrm{R}}/S}\sqrt{1+T_{\mathrm{M}}/S}}.$$

Thus the proportion of the shared variants among all variants affecting reading (and similarly for mathmetics) is

$$\frac{S}{S+T_{\mathrm{R}}} = (1 + T_{\mathrm{M}}/S)\left(\frac{\rho_G}{\rho_S}\right)^2 \geq \rho_G^2.$$

By plugging in the point estimates of 0.74 (population level analysis) and 0.64 (twin analysis) for ρ_G we get lower bounds of 0.55 and 0.41 for the proportion of shared variants for the traits. We note that if we further assume that $T_{\mathrm{R}} = T_{\mathrm{M}}$, that is, both traits are controlled by a similar number of variants, then the lower bound raises from ρ_G^2 to ρ_G which leads to bounding the proportion of shared variants from below by 0.74 and 0.64 for population level and twin analysis, respectively.

Decomposing correlation. In the main text we state that at least around a half of the observed correlation in reading and mathematics abilities is due to underlying genetics. To explain this, we write for the population level model

$$\begin{aligned}
\mathrm{Cor}(Y_i^{\mathrm{R}}, Y_i^{\mathrm{M}}) &= \frac{\mathrm{Cov}(G_i^{\mathrm{R}} + \varepsilon_i^{\mathrm{R}}, G_i^{\mathrm{M}} + \varepsilon_i^{\mathrm{M}})}{\sqrt{V^{\mathrm{R}}V^{\mathrm{M}}}} = \frac{\mathrm{Cov}(G_i^{\mathrm{R}}, G_i^{\mathrm{M}}) + \mathrm{Cov}(\varepsilon_i^{\mathrm{R}}, \varepsilon_i^{\mathrm{M}})}{\sqrt{V^{\mathrm{R}}V^{\mathrm{M}}}} \\
&= \frac{\mathrm{Cov}(G_i^{\mathrm{R}}, G_i^{\mathrm{M}})}{\sqrt{V^{\mathrm{R}}V^{\mathrm{M}}}} + \frac{\mathrm{Cov}(\varepsilon_i^{\mathrm{R}}, \varepsilon_i^{\mathrm{M}})}{\sqrt{V^{\mathrm{R}}V^{\mathrm{M}}}} \\
&= \rho_G\sqrt{\frac{V_G^{\mathrm{R}}}{V^{\mathrm{R}}}\frac{V_G^{\mathrm{M}}}{V^{\mathrm{M}}}} + \rho_\varepsilon\sqrt{\frac{V_\varepsilon^{\mathrm{R}}}{V^{\mathrm{R}}}\frac{V_\varepsilon^{\mathrm{M}}}{V^{\mathrm{M}}}},
\end{aligned}$$

where the first term is the genetic contribution to the correlation and the second term is the contribution of the error terms. Using the population level point estimates of the parameters we have that the genetic contribution to the correlation is $0.28 = 0.74\sqrt{0.27 \times 0.52}$ and the error part is $0.32 = 0.54\sqrt{0.73 \times 0.48}$. Thus the proportion of the observed correlation that is due to (additive) genetics (tagged by available SNPs) is $0.47 = 0.28/0.60$. By applying the same formula to the twin analysis, the corresponding quantity is $0.62 = 0.37/0.60$.

Supplementary References

1. Meaburn EL, Harlaar N, Craig IW, Schalkwyk LC, Plomin R. Quantitative trait locus association scan of early reading disability and ability using pooled DNA and 100K SNP microarrays in a sample of 5760 children. *Mol. Psychiatr*. **13**, 729–40 (2007).

2. Scerri TS, Morris AP, Buckingham L-L, Newbury DF, Miller LL, Monaco AP, et al. DCDC2, KIAA0319 and CMIP Are Associated with Reading-Related Traits. *Biological Psychiatry* **70**, 237–45 (2011).

3. Poelmans G, Buitelaar JK, Pauls DL, Franke B. A theoretical molecular network for dyslexia: integrating available genetic findings. *Mol Psychiatr*. **16**, 365–82. (2011).

4. Docherty SJ, Davis OSP, Kovas Y, Meaburn EL, Dale PS, Petrill SA, et al. A genome-wide association study identifies multiple loci associated with mathematics ability and disability. *Genes Brain Behav*. **9**, 234–47. (2010).

5. Haworth, C.M.A., Davis, O.S.P. & Plomin, R. Twins Early Development Study (TEDS): A Genetically Sensitive Investigation of Cognitive and Behavioral Development From Childhood to Young Adulthood. *Twin research and human genetics : the official journal of the International Society for Twin Studies* **16**, 117-25 (2013).

6. Kovas, Y., Haworth, C.M.A., Dale, P.S. & Plomin, R. The genetic and environmental origins of learning abilities and disabilities in the early school years. *Monographs of the Society for Research in Child Development* **72**, vii, 1-144 (2007).

7. Markwardt, F. C. Jr. P*eabody Individual Achievement Test—Revised (normative update) manual.* Circle Pines, MN: American Guidance Service (1997).

8. GOAL plc. *GOAL formative assessment: Key stage 3.* London: Hodder & Stoughton (2002).

9. Woodcock, R. W., McGrew, K. S., & Mather, N. *Woodcock-Johnson III Tests of Achievement.* Itasca, IL: Riverside Publishing (2001).

10. Torgesen, J. K., Wagner, R. K., & Rashotte, C. A. *Test of Word Reading Efficiency.* Austin, TX: Pro-Ed (1999).

11. NferNelson Publishing Co. Ltd. *Mathematics 5–14 series.* Windsor, UK: Author (1999).

12. Boker S., Neale M., Maes H., Wilde M, Spiegel M., Brick T., Spies J., Estabrook R., Kenny S., Bates T., Fox J.: OpenMx: An open source extended structural equation modelling framework, *Psychometrika* **76** 306-317 (2011)

13. Deary I. J., Yang J, Davies G., Harris S. E., Tenesa A., Liewald D., Luciano M., Lopez L.M. Gow A.J., Corley J., Redmond P., Fox H.C., Rowe S.J., Haggarty P., McNeill G., Goddard M.E., Porteous D.J., Whalley L.J., Starr J.M., Visscher P.M.: Genetic contributions to stability and change in intelligence from childhood to old age, *Nature* **482** 212-215 (2012)

14. Korte A., Vilhjalmsson B.J., Segura V., Platt A., Long Q., Nordberg M.: A mixed-model approach for genome-wide association studies of correlated traits in structured populations, *Nat. Genetic.* **44** 1066-1071 (2012)

15. Lynch M., Walsh B.: Genetics and Analysis of Quantitative Traits, *Sinauer Associated Inc. USA* (1998)

16. O'Hagan A., Forster J: Kendall's Advanced Theory of Statistics. Vol. 2B. Bayesian Inference, *Arnold, London* 2nd edition (2004)

17. Pirinen M., Donnelly P., Spencer C.: Efficient computation with a linear mixed model on large-scale data sets with applications to genetic studies, *Ann. Appl. Stat* **7** 369-390 (2013)

18. Visscher P.M., Yang J., Goddard M.E.: A commentary on "Common SNPs explain a large proportion of the heritability for human height" by Yang et al (2010), *Twin Research and Human Genetics* **13** 517-524 (2010)

19. Yang J., Benyamin B., McEvoy B.P., Gordon S., Henders A.K., Nyholt D.R., Madden P.A., Heath A.C., Martin N.G., Montgomery G.W., Goddard M.E., Visscher P.M.: Common SNPs explain a large proportion of the heritability for human height, *Nat. Genet.* **42** 565-569 (2010)

20. Yang J., Lee S.H., Goddard M.E., Visscher P.M.:GCTA: a tool for genome-wide complex trait analysis, *Am. J. Hum. Genet.* **88** 76-82 (2011)

21. Zaitlen N., Kraft P.: Heritability in the genome-wide association era, *Hum. Genet.* **131** 1655-1664 (2012)

22. Zuk O., Hechter E., Sunyaev S.R., Lander E.S, The mystery of missing heritability: Genetic interactions create phantom heritability, *Proc. Natl. Acad. Sci. USA* **109** 1193-1198 (2012)
